# The multifaceted role of mitochondria in cardiac function: insights and approaches

**DOI:** 10.1186/s12964-024-01899-x

**Published:** 2024-10-29

**Authors:** Sriram Ravindran, Christoph D. Rau

**Affiliations:** Computational Medicine Program, Department of Genetics, and McAllister Heart Institute, University of North Carolina at Chapel Hill, 116 Manning Drive, Chapel Hill, NC-27599 USA

**Keywords:** Cardiovascular disease, Organellogenesis, Mitochondrial subpopulations, Cell-organelle communication, Computational biology, Mitochondrial genetics

## Abstract

Cardiovascular disease (CVD) remains a global economic burden even in the 21st century with 85% of deaths resulting from heart attacks. Despite efforts in reducing the risk factors, and enhancing pharmacotherapeutic strategies, challenges persist in early identification of disease progression and functional recovery of damaged hearts. Targeting mitochondrial dysfunction, a key player in the pathogenesis of CVD has been less successful due to its role in other coexisting diseases. Additionally, it is the only organelle with an agathokakological function that is a remedy and a poison for the cell. In this review, we describe the origins of cardiac mitochondria and the role of heteroplasmy and mitochondrial subpopulations namely the interfibrillar, subsarcolemmal, perinuclear, and intranuclear mitochondria in maintaining cardiac function and in disease-associated remodeling. The cumulative evidence of mitochondrial retrograde communication with the nucleus is addressed, highlighting the need to study the genotype-phenotype relationships of specific organelle functions with CVD by using approaches like genome-wide association study (GWAS). Finally, we discuss the practicality of computational methods combined with single-cell sequencing technologies to address the challenges of genetic screening in the identification of heteroplasmy and contributory genes towards CVD.

**Supplementary Information:**

The online version contains supplementary material available at 10.1186/s12964-024-01899-x.

## Background

 The recently concluded Wellcome Conference on Mitochondrial Medicine (2024) held in the UK brought together world leaders to discuss the advances and voids in mitochondrial disease research [1]. Keynote speakers highlighted the need to depart from traditional therapies addressing biochemical changes and adopt strategies to replace mutant mitochondria, specific to the disease and organ affected. However, such targeted approaches need well-characterized models as the same mitochondrial genetic variations may express different phenotypes across individuals, making identification of the best therapeutic course of action a challenge in many studies. Advanced technologies in sequencing identifying the causal variants, gene therapies, and large human trial data need to be utilized for this purpose. Likewise, the National Heart, Lung, and Blood Institute’s (MD, USA) expert group on Mitochondrial research in the Cardiovascular system has highlighted the lack of rigorous and quantitative information in the field to explore mitochondrial therapies that can treat heart failure (HF) [2]. The primary recommendation of the group was to foster a cross-disciplinary approach by bringing together Biologists, Bioinformaticians, Engineers, and Clinicians in designing team science proposals to understand mitochondrial functions. To do so, there needs to be a comprehensive review covering the current knowledge and systems-based approaches that can help researchers in multidisciplinary teams identify promising future directions. Our review covers fundamental aspects of mitochondria in cardiac development, its role in pathology, repair, and remodeling along with the various signaling pathways involved in the quality control of this energetic organelle. However, it is incomplete without providing solutions that answer many of the key questions that remain elusive in the community such as the role of distinct sub-populations of mitochondria in cardiomyocytes, the contribution of mitochondrial DNA (mtDNA) variants, and the effects of mitochondria-nucleus crosstalk to cardiovascular health. Therefore, we discuss some of the existing state-of-the-art tools available to the community to provide computational rigor in unraveling some of these mysteries, benefit from the data available in some of the repositories, and generate new approaches to study the role of mitochondria in cardiovascular disease. In recent times, several genomic technologies such as long-range sequencing, single-cell RNAseq, deep-sequencing etc., have made it possible to study the organelle in extreme detail and the data generated should not remain underutilized by multidisciplinary groups. Our aim of the review is to bring these groups together to study this organelle and design therapeutic approaches that would treat cardiovascular disease.

## Mitochondria in cardiac development

### Respiratory transitions in cardiogenesis

Cardiogenesis is a well-orchestrated event beginning from the heart tube formation to the first beat of the embryo at 3 weeks into gestation in humans. The fascinating arrangement of complex cell types in the heart arises from the maturation of progenitor cells in the fertilized egg from the cardiac mesoderm, proepicardium, and cardiac neural crest [3]. Given the hypoxic environment in which embryogenesis occurs, meeting the energy requirements of the cell is critical in determining the growth and fate of the embryo. Hence it is not surprising to note that 100,000 mitochondria are harbored in the oocytes compared to 5000–8000 in an adult cardiomyocyte [4, 5]. Rather, the metabolic transition in embryogenesis is intricate and involves signaling molecules that drive the differentiation and transition processes as outlined in Table 1. Disturbances in the transition process as observed during preterm birth predispose individuals to cardiovascular-related mortality in adulthood [6].

**Table 1:** Metabolic transitions in cardiogenesis and re-distribution of mitochondria

Cell type	Respiration	Energy substrate	Mitochondrial distribution and features	Ref.
Oocyte	anaerobic	Pyruvate > Lactate > fatty acid > Glucose	*Distribution*: Structured subpopulations of mitochondria exist with 90% of the ooplasm occupied by mitochondria mostly distributed in the perinuclear region. They do not exhibit usual morphology and have arc-like cristae *Features*: Even in presence of oxygen and functional mitochondria, oocyte utilizes lactate/pyruvate for energy by PPP rather than glucose by glycolysis because the energy is instant. Use of the adenosine salvage pathway was found prominent in case of bovine oocyte	[7–11]
Blastocyst	aerobic (TE cells) and anaerobic/quiescent (ICM)	Glucose > glutamate > Pyruvate/Lactate > Fatty acids	*Distribution*: During late morula, mitochondria appear vesicular with sparse cristae due to anaerobic glycolysis and gradually transform to a lamellated form with the maturation of embryo to accommodate ETC and start with the aerobic oxidation but have immature cristae. In TE mitochondria are long and slender while the ICM mitochondria are spherical due to greater oxygen consumption by TE compared to ICM *Features: A*ccumulation of mitochondria around ER is a feature observed in this stage. Rapid increase in oxygen consumption compared to the single cell zygote stage. The vesicular state also prevents accumulation of toxic ROS products. At implantation, first the trophectoderm and then the entire embryo gain capacity to replicate mtDNA. The mtDNA replication does not take place up to and including the morula stage and the number of mtDNA copies/blastomere is progressively decreased after each embryonic cell division. This results in these blastomeres progressively losing their capacity to generate ATP through OXPHOS as they become more reliant on anaerobic respiration	[11–14]
Mesoderm (Lateral plate mesoderm > Cardiogenic mesoderm > heart fields)	High dependence on OXPHOS (*Key point of metabolic switch with highest basal RCR*)	Pyruvate/Glutamate	*Distribution*: Mitochondria transform from a granular and fragmented structure to a filamentous reticular elongated network with mature cristae that can produce energy more effectively *Features*: Mitochondrial fission is inhibited at this stage to conserve and produce more energy and less ROS. Despite reduction in mitochondrial mass and mtDNA levels, cells show high mitochondrial efficiency The Drp1 gene silencing induces metabolic switch with reliance on anaerobic glycolysis	[15, 16]
Endoderm	Dependent on OXPHOS	Glucose	*Distribution*: Mitochondria are elongated and matured as they differentiate from mesoendoderm to endoderm and show high respiratory capacity Most studies are reported in stem cells and results in vivo systems is not known	[17, 18]
Cardiac neural crest	aerobic glycolysis and low OXPHOS	Glucose > cholesterol (Shh pathway)	*Features*: Mitochondria are responsible for differentiation of neural crest cells [19]. metabolic abnormalities leading to neurocristopathy is the reason behind Leigh syndrome	[20]
Proepicardium/epicardium	Aerobic	Glucose	*Features*: Undergo mesenchymal to epithelial transformation to form the epicardium protecting the heart	[21]

*PPP* pentose phosphate pathway, *ETC* electron transport chain, *RCR* respiratory control ratio, *TE* trophoectoderm, *ICM* Inner cell mass, *ER* Endoplasmic reticulum, *Shh* Sonic hedgehog pathway

**Fig. 1:**
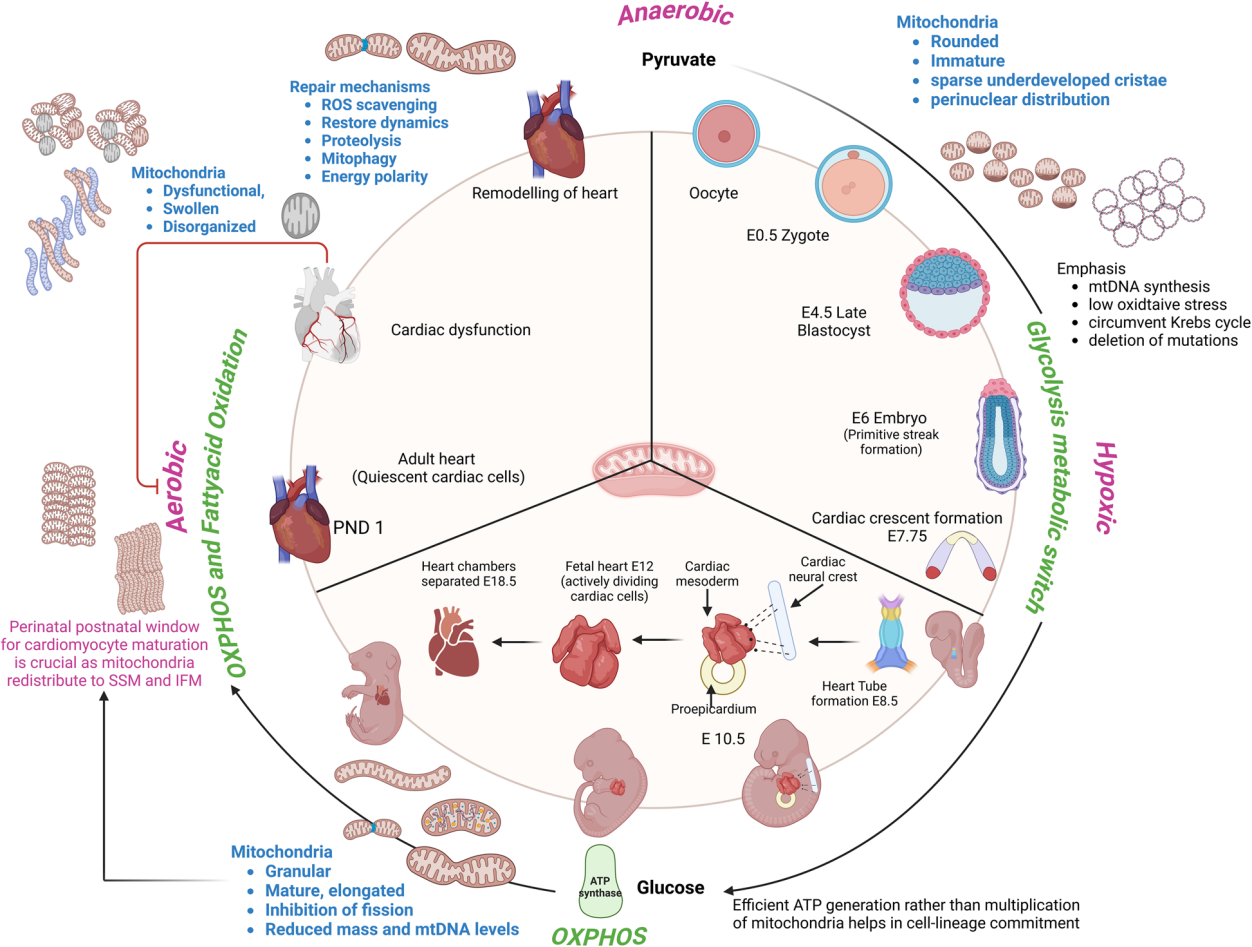
A visualized summary of mitochondrial participation in cardiac development

The transition from an oocyte to fetal, neonatal, and adult is immensely complex and involves a metabolic phase transition from hypoxic to anoxic to aerobic environments linked to a shift from glycolysis to fatty acid oxidation [7, 8, 22]. Figure 1 summarizes the key aspects of mitochondrial participation in the course of this cardiac development phase. During this phase transition, the mitochondria actively provide energy to the cell while simultaneously undergoing a dramatic transition from an immature, rounded organelle with sparsely developed cristae to a mature, organized, and elongated structure with dense cristae architecture [12, 17, 22]. Recently Beutner et al. (2024) compared mitochondria from myocytes of mouse embryonic hearts at different intracellular locations and confirmed that location dictates mitochondrial structure and function [23]. While the known spatially distinct populations in an adult heart are interfibrillar, subsarcolemmal, and perinuclear, the study defined a new “cytoplasmic” category to differentiate the predominant mitochondria found during the embryonic stages (E9.5) that are not associated with any particular structure, and that disappear by post-natal day1. The perinatal window is the critical stage during which changes to mitochondria structure and distribution increase the mitochondrial volume in myocytes. Interfibrillar and subsarcolemmal subtypes are localized predominantly in the postnatal myocytes while perinuclear mitochondria support the embryo predominantly at E16.5. To accommodate the structural maturation, more copies of mtDNA are made to promote the electron transport chain (ETC) gene expression, supporting the increasing demands of the developing heart. The hypoxic conditions help in mtDNA synthesis and avoid oxidative stress in the immature mitochondria by circumventing the Krebs cycle. Oocytes have an evolutionarily conserved strategy of suppressing the complex-I function to maintain a ROS-free environment for healthy embryogenesis [24]. Next, the shift in energy substrate from pyruvate to glucose (10–85%) during the development of blastocyst leads to a metabolic switch to oxidative phosphorylation (OXPHOS) to generate adenosine triphosphate (ATP) more efficiently as the number of mitochondria per cell is diminished. A plausible explanation for such adaptations is that these processes reduce stress-induced mutations in mtDNA, which is important as they lack protective histones or repair mechanisms. Thus, mtDNA is highly stable, and pathological mutations are selectively eliminated before embryogenesis in a process called purifying selection [25]. Abnormalities in glucose metabolism/utilization at this stage have resulted in physiological complications due to abnormal mitochondrial energy production and division [26]. On the contrary, supplementation of oocytes with autologous mitochondria resulted in compromised cardiac architecture (mucoid degeneration of the valves) and weight gain in the offspring despite enhancing fertility and thereby raises questions about the use of the technique for assisted reproductive technologies [27]. Arribat et al., (2019) demonstrated in mice that the epigenetic influence of the additional copies of mtDNA on the nuclear DNA (nDNA) resulted in the cardiac abnormalities highlighting the tight regulation of mitochondrial quality control and the organelle-organ communication [28]. Further post-natal stages show a rapid conversion to fatty acid oxidation (80%) from glucose owing to its increased uptake by the mitochondria, activated by several key regulators of the tricarboxylic acid (TCA) cycle and the availability of oxygen [29, 30]. Although metabolic switching seems beneficial, it is identified as the key perpetrator of regeneration as the process was found reversible by inhibition of *Pdk4*, which enhances glucose oxidation [31, 32]. Therefore, for grasping the mitochondria’s involvement in cardiomyocyte maturation and regeneration under pathological conditions, it is imperative to understand the transcriptional signals and inter-organelle communications that drive the process.

### Mitochondrial adaptations and their impact on cardiogenesis

Mitochondria play a critical role in eukaryotic cell differentiation. Typically, the immature mitochondria in stem cells, influenced by metabolic transitions, develop complex structures, propelling the organelle to undergo fission, a prerequisite for differentiation [33]. The process is triggered by the Wnt and Notch signaling pathways through the Forkhead box O (FoxO) transcription factors that control the mitochondrial fission-1 (Fis1) gene. Contrary to this hypothesis, Lee (2020) found that the morphological changes in mitochondria that could trigger differentiation were discrete from the expression of Fis1 and depended on a balance between fusion and fission as explained by the ratio of genes mitofusion2(Mfn2) and dynamin-1-like (Dnm1L) [34]. An in-depth understanding of the process of organogenesis and organellogenesis during development demonstrated that mtDNA quality control is regulated by 11 proteins (TFAM, POLG, TWINKLE, TOM complex, TIM complex, HSP60, CLPP, LONP1, OPA1, MFN1/2, and DRP1) that regulate the OXPHOS pathway [27, 28]. When mtDNA replication is promoted by lineage-specific markers (vimentin, nestin, β-tubulin), upregulation of PolgA, PolgB, Tfam, ATPase5b, COXI is observed, and the organelle differentiates to an elongated form with swollen cristae to consume more oxygen and generate higher membrane potential and ATP. This adaptation of organelle with organ goes hand-in-hand with distinct patterns of mitochondrial distribution observed in the myogenesis of zebrafish (promoted via the Sonic hedgehog pathway) and the mtDNA mosaicism (Heteroplasmy: existence of both mutant and wild-type mtDNA genomes within a cell) in human development reported recently [35].

Cardiac mesoderm is considered as the starting point of mammalian cardiogenesis. The differentiation of mesodermal cells to the heart-forming area is controlled by signaling molecules involved in the Wnt/β-catenin pathway, TGFβ and BMP2. Several detailed reports of the process and commitment to various cell types and their contribution to portions of the heart have been presented in previous reviews [36, 37]. Here we emphasize the role of mitochondria to the development process as there are recent fascinating reports about the ways in which mitochondrial dynamics are involved in cardiogenesis [15, 38]. Mostafavi (2021), observed that despite higher mitochondrial activity, coupling efficiency and higher ATP production during mesodermal differentiation, there was a decrease in organelle content and its DNA compared to undifferentiated human pluripotent stem cells (PSCs) [38]. This could be caused by a process of morphological adaptation to drive cell-lineage commitment rather than multiplication as demonstrated earlier in induced PSCs. Involvement of mitochondrial fission gene Drp1 was found to limit the mesodermal differentiation as in this case, the use of Mdivi-1 a selective Drp1 inhibitor elevated the mesodermal expression of cardiac genes in the stem cells accompanied by increased respiratory activity in the mitochondria and decreased glycolysis. In a recent similar study, depletion of Tfam also reduced mesoderm differentiation efficiency and the commitment of human PSCs to cell-lineages highlighting the regulatory role of mitochondria in cell-fate determination and demonstrating that any defects in the process could lead to fetal cardiac abnormalities [39].

Evidence suggests that the maturation of cardiomyocytes is driven in part by changes in the distribution of mitochondria among its subcellular locations, notably the subsarcolemmal and interfibrillar populations [40]. Cardiac cells transition from an actively dividing state in the fetus to a quiescent state postnatally. Their independent respiratory ability is supported by well-developed mitochondria which support the heart with the energy required for its uninterrupted contractility. The perinatal-postnatal window is a crucial phase of cardiomyocyte maturation and mitochondria contribute to the maturation process as they move from perinuclear spaces to interfibrillar and subsarcolemmal spaces with designated functional and biochemical independency [22]. The interfibrillar mitochondria (IFM) has higher ETC activity compared to the subsarcolemmal mitochondria (SSM) and supports energy for contractile activity while the SSM provides energy to support the biochemical reactions in the muscle cells [41]. Although maturation of cardiomyocytes (CMs) and mitochondria are intertwined, there is evidence to suggest that mitochondria respond to metabolic stimuli first and drive subsequent cell maturation [30]. First, stimulation by the hypoxic environment drives the perinuclear distribution of mitochondria and fission-fusion dynamics through AMPK, HIF-1α and mTOR signaling pathways. Second, the availability of several metabolic drivers to generate ATP such as glucose, fatty acids, branched-chain amino acids (BCAA), hormones, glucocorticoids, estrogen, retinoic acid, angiotensin-II, Noncoding RNA and O-GlcNAcylation, drive the crosstalk between genetic and metabolic signaling [40]. The fetal dependence on glycolysis shifts to OXPHOS post-natally from breast milk which contains higher amounts of fatty acids [42]. This is associated with the maturation of CMs. Recent empirical evidence by Li (2023) confirmed that activation of AMPK is a driving factor for the maturation of CMs derived from human-induced PSC’s preceded by mitochondrial maturation [43]. Activating AMPK increased cellular ATP, promoted biogenesis, increased the membrane potential and fatty acid uptake, clearly indicating metabolically mature organelle drive cardiomyocyte development. Thus, the maturation of mitochondria may not be a consequence of cardiomyocyte maturation but may actually trigger the maturation due to their crucial function in maintaining the metabolic phenotype [44]. The process of postnatal heart development needs to be systematically studied because nutrient changes during the first few days after birth may have significant effects on mitochondrial structure, physiology, biochemical processes and regulate the cell cycle.

### Inter-organelle communication and the role of mitochondria

During development, particularly in the perinatal-postnatal window, changing metabolic environments prompt dynamic interactions among cell organelles [45]. Contact sites between organelles are extremely close (~ 10 nm) and allow for efficient signaling with loss of contact known to cause autophagic responses. Among these communications, mitochondria play an essential role as they occupy 35% of the cell volume of an adult cardiomyocyte and are responsible for handling the high energy demands of the heart and calcium dynamics required for rhythmic electrical activity. Hence it is not surprising that imbalances in such inter-organelle communication in the heart frequently result in pathological conditions. More importantly, these could be divided into two aspects (a) mitochondria-nuclear communication and (b) mitochondria-cell organelle communication (mainly endoplasmic reticulum (ER), Golgi, and lysosome). Here, we focus on the role of mitochondria-organelle crosstalk in the context of cardiac development and the healthy heart.

### Mitochondria communication with the nucleus

Unknown until 1991, mitochondrial communication with the nucleus has been recognized as a key process to maintain organelle homeostasis in yeast [46, 47]. Butow and Avadhani [45] were the earliest to investigate the mito-nuclear signaling mechanism in yeast models & mammalian cells, identifying the key regulatory factors converging at TOR signaling in the former while it was Ca^2+^ dynamics in the latter. In order to control its own metabolism, physiological functions, and respond to stress stimuli, mitochondria have to signal the nucleus to regulate relevant genes by communicating through retrograde signaling [48]. Physically mitochondria are known to interact with the other organelle via MAM (mitochondria associate membranes) [49, 50]. They help to regulate calcium, lipid and intracellular trafficking and include multiple protein complexes & ion channels. Many such ‘Mitonuclear’ communications have been reviewed by Quirós et al. and are essential for orchestrating cellular events [51]. The complex interplay however needs additional evaluation through in vivo models at different genetic scales, to understand this complex regulation during heart development. Despite the fascinating ways by which the mitochondria communicate with the nucleus, direct nucleus-mitochondrial contact has not been reported yet [52].

Berg and Kurland hypothesized that the retrograde translocation of genes to the nucleus is purely based on selection and high mutation in mitochondrial genes under stress [53]. The emerging reports on carrier-mediated translocation of mitochondrial proteins under stressed environment has proven the hypothesis with identification of GPS2 protein as an intermediary for mitochondrial retrograde signaling that can activate nuclear-encoded mitochondrial genes and mediate translocation of AIFm2 (primarily located in the mitochondrial intermembrane space) to nucleus by forming HNE adducts (transport mechanisms specific for translocation) only under pathophysiological states [54–56]. A mouse model of cardiomyocyte-specific knockout of MnSOD was the first evidence of embryonic lethality caused by the translocation of AIFm2 to the nucleus resulting in apoptosis [57]. As the nuclear effectors of mitochondrial signaling are well-known to control the quality of the organelle, mitochondrial signaling is known to help the cell eliminate dysfunctional organelles and is distinctly bidirectional based on stress responses. To date, some of the canonical signals include calcium, ROS, Adenine nucleotides (ADP, AMP, NAD, NADH) and TCA intermediates (Acetyl-CoA, αKG, succinate, fumarate) which have been studied extensively in yeast systems [58]. Though several studies have evaluated signaling proteins that function retrogradely [59], there are very few studies that specifically identified the role of retrograde signaling in the mammalian heart and have been listed in Table 2. Novel techniques such as in-situ mapping of RNA-genome interactions lately provided evidence of a previously unidentified phenomenon of transcriptional regulation of nuclear signaling by the mitochondria in endothelial cells [57]. The study revealed an increasing association of mitochondrial RNA with nuclear chromatin in diabetic stress, resulting in the activation of inflammatory responses. A compelling model in the mammalian system has been developed recently with mice engineered to have different mtDNA backgrounds that showed differences in cardiac metabolism and aging [60]. The increased non-pathogenic heteroplasmy in this model caused progressive metabolic stress during adulthood possibly due to nuclear-mitochondrial cross-talk. Such models hold promise for future studies to explore inter-organelle communication. Computational advancements from Hodgkinson [51], Ryten [61], and Chiu [62] labs hold promising approaches for the future to decipher the role of retrograde signaling in cardiac development. Several molecular mechanisms in this area have been studied only in yeast systems, whose role needs evaluation in the development of the developing heart.

**Table 2:** Identified roles of retrograde (bi-directional) signals that control mitochondrial-nuclear signaling established in heart

Retrograde signal	Role in cardiac physiology	Ref.
p53	Cause stress induced apoptosis. Absence of p53 reduced mitochondrial injury and improved cardiac function	[63]
AIFm2	Translocation induces apoptosis	[55, 56]
SP1	Calcium regulation	[64]
SIRT3	Cardioprotective in stress induced cell death	[65]
miR-378	Cardioprotective antiapoptotic miRNA	[66]
HIG2A	Resistance to hypoxia	[67, 68]
UPR^mt^	Stress response to recover defective mitochondria (Critical pathways involved: a) mtHSP70 and HSF1, b) ER*α*-NRF1-HTRA2, c) ATF4/ATF5-CHOP, Sirt3-FOXO3a-SOD2)	[69, 70]
Atfs-1	Not known	-
CLK-1	Not known	-
Rtg1P/Rtg3P	Not known	-
Fumarase	Elevated fumarate is Cardioprotective via activation of the Nrf2 Antioxidant Pathway	[71]
TIN2	Not known	-
RECQL4	Not known	-

Contrary to the belief that the mammalian mtDNA is a naked, circular, unshielded structure with multiple individual copies in an organelle, it is actually packaged into heritable units of distinct ellipsoid mtDNA-protein complexes consisting of multiple mtDNA copies termed as the ‘mitochondrial nucleoid’ [72]. Because of its compact shape, lack of introns, and frequent overlapping of reading frames between nearby genes, mtDNA has a higher gene density than nDNA [46]. Despite its small size, mtDNA-transcribed mRNA has a remarkable presence across the total cardiac mRNA (30%) [47]. The mitochondrial nucleoid is composed of more than 50 proteins that provide stability to the mtDNA much like histones with nuclear DNA. Of critical importance among these proteins is Tfam, which maintains the packaging, replication, copy number and transmission of mtDNA. Several studies have reported that inactivation of Tfam caused lethality in embryonic and adult hearts characterized by myocardial wall thickening, dilated cardiomyopathy, and mitochondrial dysfunction [73, 74]. Recently, the presence of nuclear-encoded transcription factors (MOF, AP1, CEBPB, MEF2D), and the contribution of nuclear spliceosome complex to mtDNA-splicing provided strong evidence of control of mitochondrial replication/transcription demonstrating evolutionary adaptation of the mammalian mtDNA in the nucleoid [75]. Apart from the proteins forming the mitochondrial nucleoid, the mitochondria are regulated by the non-coding RNA that contributes to cardiac homeostasis. Most widely studied among them are the microRNAs termed as ‘mito-miRs’ which have a preferential mitochondrial localization. Although their origin has been debated considering the presence of pre-miRNA in mitochondria which suggests their origin from mtDNA, their expression has been critical in regulation of OXPHOS in infarcted hearts (miR-1 and miR181c) [76]. The import-export mechanism for these small non-coding RNA are topics of ongoing research to reveal the nuclear-mitochondrial crosstalk. Likewise, mitochondria lack import for long non-coding RNAs and their presence within the organelle could well direct their origins to mtDNA. The LIPCAR (long noncoding RNA uc022bqs.1) has been the only mitochondrial long noncoding RNA upregulated in patients with LV remodelling following MI [77]. In conjunction, evidence of circular RNAs in the mitochondria could be the early evidence for nuclear-mitochondrial crosstalk [78] but their cardiac-specific roles are yet to be uncovered. However, these could be of potential diagnostic or therapeutic use as suppressing the mitochondrial fission and apoptosis-related circular RNA (MFACR), the first circular RNA to be discovered in the heart, was found to be cardioprotective in mice. MFACR suppression resulted in reduced mitochondrial fission mediated by the interaction of miR-652-3p and *MTP18* [79].

### Mitochondria communication with other organelles

The positioning of organelles in heart muscle cells is governed by tightly regulated processes such as Ca^2+^ regulation, autophagy, and lipid metabolism. Through early electron-microscopy studies on embryonic heart development, we know that the myocardium is not a uniform compact tissue and that myocytes contain scattered free ribosomes, Golgi, mitochondria, granular endoplasmic reticulum, and phagocytes. Progressive development brings order and differentiation to heart tissue. The proteins required for the recruitment of additional cell types to the developing heart are processed by these organelles to create extra-cellular matrix and collagen and the development of additional layers of fibroblasts [48]. It is this reorganization phase that necessitates contact among organelles to meet the requirements of cardiomyocyte function [52]. While it is known that embryonic development is accompanied by changes in the volumes of organelles, we lack information on how these organelles communicate to maintain their volumes according to cellular requirements [45].

Recently, Kim (2022) mapped physical interactions at high resolution between mitochondria, sarcoplasmic reticulum, and lipid droplets in mice hearts using focused ion beam scanning electron microscopy. The images showed the highest surface area interaction by postnatal day 14. This is supported by the fact that postnatal cardiomyocyte contraction is accompanied by the maturation of Sarcoendoplasmic Reticulum Calcium ATPase (SERCA) pumps by day 21 [80]. Any altercation of these interactions can severely affect the heart function. In 2023, Jan Boren’s group demonstrated that PCSK9-deficient mice had impaired contractile function leading to cardiomyopathy and premature death due to altered mitochondria-ER contact [81]. This is also the case in Mfn2 deficient mice (Mfn2 tethers mitochondria to ER) [82]. A more direct contact with ER was established by FUNDC1, an outer mitochondrial membrane protein, by binding to the Inositol triphosphate (IP3) receptor which was significantly suppressed in patients with HF [83]. Thus, cardiac remodeling during postnatal development is accompanied by organelle interactions which is critical for calcium handling and energy distribution. A comparison of interactions across mitochondrial subtypes has not been made yet that would reveal developmental profiles that vary across regions where these organelles are distributed.

The mitochondria and Golgi positioning in cardiomyocyte development is a topic of interest and needs more research as they complement each other by acting as Ca^2+^ sinks and regulating protein trafficking. Given the highly dynamic environment of the embryo, Yuri Morozov recently found that stress impacts the Golgi apparatus even before structural changes can manifest in the mitochondria [84]. The impact of an early malfunction in the Golgi on the mitochondria has not been investigated particularly in the heart although upcoming reports hint at the control of mitochondrial plasticity through Golgi network vesicles, which is interesting to study in heart development [85, 86].

Lysosomes traditionally facilitate the degradation and recycling of cellular waste through autophagy. Canonically, this process also maintains mitochondrial quality in the cell by selective removal of damaged mitochondria, called mitophagy. A recent review has sufficiently summarized the pathways leading to mitophagy and its role in cardioprotection. However, most of these reported signals are transmitted during stress or from damaged mitochondria [87]. We thereby sought to identify driving factors in cardiac developmental stress that promote mitochondria-lysosome communication. Gong et al., provided strong evidence of mitochondrial maturation in perinatal stages driven by Parkin-dependent mitophagy [88]. The increased oxygenation drives Mfn2-Parkin interaction to remove fetal mitochondria causing a metabolic shift from glycolytic to fatty acid metabolism in the mouse heart. The process is likely driven by the loss of mitochondria-ER contact sites and the recruitment of optineurin that drives the binding of autophagosome protein LC3 forming a mitophagy complex around the mitochondria [89, 90]. Studies on such mitochondria-lysosome communication are rudimentary and require an in-depth understanding of how they regulate cardiac maturation. Besides mitophagy, new roles for mitochondria-lysosomes interactions have been identified such as controlling calcium dynamics, forming hybrid organelle, and compartment-selective microautophagy [91–93]. However, their activation or role in embryonic heart development is yet to be explored.

## Mitochondrial dysfunction and cardiac remodeling

### Organelle phenotype and cardiac physiology

**Fig. 2:**
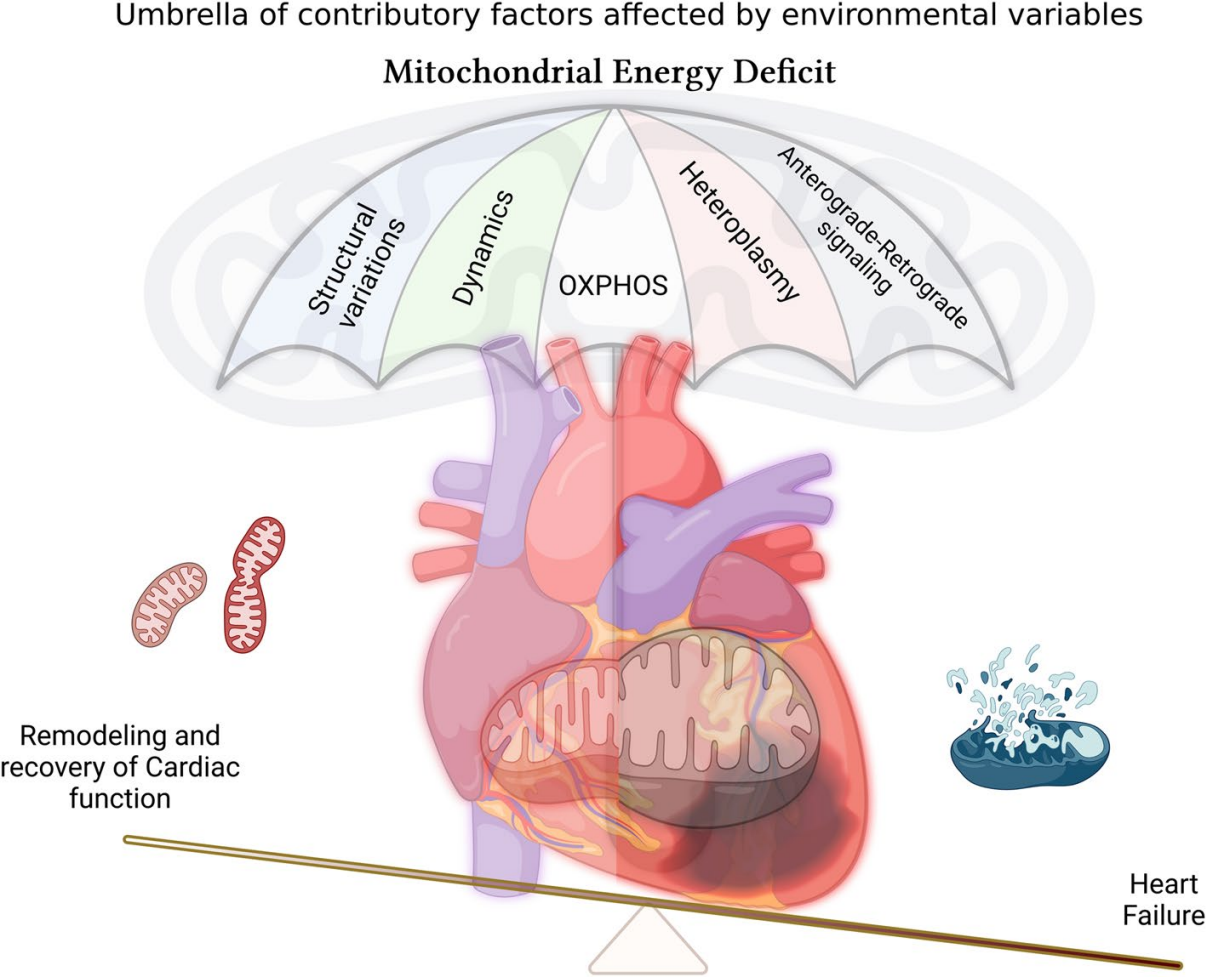
The image portrays key factors contributing to the mitochondrial health which in-turn affect the cardiac function

Early death in children with inherited mitochondrial diseases often involves cardiovascular complications which in part is due to the dysfunctional organelle contributing to the disease phenotype [94–96]. Abnormalities in mitochondria can significantly contribute to impaired OXPHOS deficiency leading to lower oxygen utilization and thereby contractile dysfunction, apoptosis, and cell death. To understand the holistic impact, studies must integrate cardiac physiology and structural alterations with mitochondrial attributes such as structure, respiratory capacity, and dynamics. When combined with integrating the consequence with genomic changes such as expression or mutation, it provides an enormous understanding of the mitochondrial basis for cardiovascular disease. Unfortunately, few studies (mostly pre-clinical) have recognized such integration to provide a comprehensive understanding of how an organelle dysfunction leads to phenotypic changes and/or remodeling (Additional Table 1 and Fig. 2). The etiology of mitochondrial dysfunction in the development of disease is complicated and is spatio-temporally associated with elevation of sympathetic signaling, oxidative stress, inflammatory response, and metabolic syndromes such as diabetes [97]. Additionally, the same mutation can lead to cardiomyopathy symptoms in some patients while others may remain asymptomatic. Current therapies targeting the reactive oxygen species (ROS), and oxidative stress were unsuccessful, exposing the dearth of knowledge we have in this field. We hereby discuss a brief outcome of studies that have integrated the heart’s function and its mitochondrial characteristics under pathological milieu (Additional Table 1). The table also provides information on attempted interventions and their targets in mitochondria.

#### Structural

Diseased hearts show distinct morphological changes in mitochondria which are in sync with functional alterations. The organelle swells due to edema, lacks cohesive cristae structure, and appears vacuolar under conditions of stress due to ischemia-reperfusion injury (IRI) [98–104], hypertrophy [105], cardiomyopathy [106–108] and acute myocardial infarction (AMI) [109, 110]. Some of these HF conditions are also encountered in comorbidities such as obesity and diabetes, which additionally contribute to the organelle’s appearance [98, 99, 111]. Notably, such hearts showed reduced ejection fraction (EF) and fractional shortening (FS) with prominent diastolic dysfunction due to stiffening without any changes in the chamber dimensions. While tachycardia and fibrillation were prominent in IRI with limited recovery of ventricular function, hypertrophy caused by diabetes resulted in reduction in E/A ratio, and left ventricular (LV) mass. The above changes were observed along with disorganized mitochondrial (irregular patterns) instead of the ‘pearl string appearance’ (linear arrangement) [99]. Interestingly, these changes did not alter the heart rate, which could have led to heart failure with preserved ejection fraction (HFpEF).

The severity of disease condition can sometimes be observed by a loss of mitochondrial number in conditions of hypoxia, and hypertrophy especially in hemodialysis and diabetes [111–113]. Quantification of the dimensions of the diseased mitochondria reveals a reduction in cross-sectional area, perimeter, and Feret diameter with increased roundedness due to IRI. These structural changes were in addition to the loss of membrane potential and lower oxygen consumption that led to premature beats.

#### Dynamics in remodeling

Cardiac stress is well-known to promote fission of mitochondria as observed in animal models of pressure overload and cardiomyopathy. Traditionally, these hearts show higher levels of fission protein DRP1 [2, 107, 114, 115]. However, this might not hold true in all cases as a recent report on neonatal cardiomyopathy revealed downregulation of fusion (*Mfn1* and *Mfn2*) and fission (*Drp1* and *Fis1*) [106]. Furthermore, diabetic hearts did not show any changes in fission/fusion protein despite the increase in area/size [113]. In addition, the study showed significant downregulation of mitofilin in the interfibrillar mitochondria from diabetic mouse hearts. These dynamic changes in cardiac mitochondria were associated with diastolic dysfunction, increased posterior wall, septum thickness along with drop in EF and FS. Recent studies indicate that mitochondrial fission is critical in promoting cardiac injury [116] and inhibition of fission using drugs (Midvi1, RTA408 and Dapagliflozin) alone was found to be cardioprotective [117–119] even in cases of comorbidities such as hypertension and diabetes [120].

#### OXPHOS

OXPHOS is critical for the supply of ATP required for myocardial contractility. Dysfunction of the apparatus leads to promotion of oxidative stress by elevating the release of free radicals. In the case of the OXPHOS, rather than changes in protein abundance, it is frequently the presence of functional mutations that disrupt the machinery. Dysfunctional / mutated OXPHOS proteins have been implicated in several mitochondrial myopathies involving mutations in genes encoding for OXPHOS proteins [96]. Although a decline in any of the complex activities means reduced respiratory capacity, reports integrating ETC-complex activity with physiology have shown innate differences across the mitochondrial subpopulations with respect to susceptibility to the pathology [115]. During pressure overload, rat hearts showed reduced complex-IV activity and uncoupling with TCA cycle but studies with an underlying condition of diabetes showed that complex I-V activities declined in IFM alone but not in SSM [113]. More recently, researchers showed that reduction in respiratory activity of IFM as a key factor for remodeling in hypertrophic cardiomyopathy in human heart tissue [121]. Given that comorbidities are a crucial aspect of CVD cases, studies in preclinical model should emphasize further studies in models with comorbidities rather than normal ones [60, 98].

#### Mitophagy

Imbalances in clearing of dysfunctional mitochondria can trigger apoptosis due to release of mitochondrial contents into the cytosol. Insufficient mitophagy is a characteristic of hearts affected by CVD and has been associated with impaired EF, FS, and high energy consumption. Autophagosomes with damaged mitochondria were a prominent observation in hypertrophic hearts which had reduced EF and increased end-diastolic left ventricular posterior wall thickness (LVPWd) without changes in LV diameter [105]. While an elevated expression of autophagy genes (*Lc3-I* and *II*,* Lamp2*,* Pink1* and *Parkin*) and proteins are featured in hypertrophy [105] and pressure-overload [115] animal models, it is more a complex phenomenon in the human heart especially in patients with diabetic cardiomyopathy (DCM) [122]. The phenotypic differences in diabetic cardiomyopathy vary across the two major types (Type1 and type2 diabetes mellitus) partially owing to opposing effects on the expression of autophagy-related pathways [123, 124]. Compelling evidence in human hearts before and after bypass surgery showed mitophagy after successful bypass which was simultaneously associated with increased biogenesis although the study lacked physiological data on cardiac function [125]. Thus, increasing turnover of mitochondria is beneficial but the differences in lab and clinical observations have posed a challenge in the translation of animal studies to patient care and should not be overlooked. Despite the differences, the characteristic physiological changes in hearts from some of these studies did not differ from those observed in hypertrophy. Rather, stabilizing functional mitochondria looks to be a promising tool in improving cardiac function associated with mitochondrial impairment [121].

#### Copy-number

Mitochondrial DNA Copy number obtained from the leukocyte is recognized as an independent predictor of CVD [126] and is inversely related to the risk of HF [127]. Recently, Genome wide association studies (GWAS) identified the influence of mtDNA on nDNA methylation through the use of multi-center datasets drawn from several major CVD trials [128]. In its simplest form, copy number is interpreted as the ratio of mtDNA/nDNA. While most clinical studies have reported this number from the blood, preclinical approaches have measured the tissue levels and found similar results. Andres et al., observed increased copy number in heart tissue perfused with blood post-cardiopulmonary bypass as being associated with better functional recovery and improved biogenesis of the organelle, missing the link with cardiac physiology [125].

Overall, in the backdrop of robust mechanisms of mitochondrial genesis and clearance, abnormalities in the mitochondria seemed to be disease and comorbidity-related. The lack of comprehensive understanding of mitochondrial physiology with cardiac physiology has made it difficult to understand CVD pathology which is why drug discovery in certain areas is lacking such as cardiac IRI and AMI. Also, differences across species and limitations of studies reasserting the results in comorbidities have made it difficult for translation to bedside. Studies aimed at co-morbidities and their influence on mitochondria in cardiac IRI were broadly reported in pre-clinical studies by the Kurian group, showing loss of cardioprotection in DCM was due to mitochondrial dysfunction [129–132]. Recently, a mouse model of non-pathologic mtDNA heteroplasmy has been developed which exhibits multiple comorbidities such as HF, pulmonary hypertension, sarcopenia, frailty and premature death in adulthood [60]. Exploring such models will redefine our understanding of the canonical signaling pathways in cardioprotection and help design and identify new drug targets.

## Mitochondria in cardiac repair

**Fig. 3:**
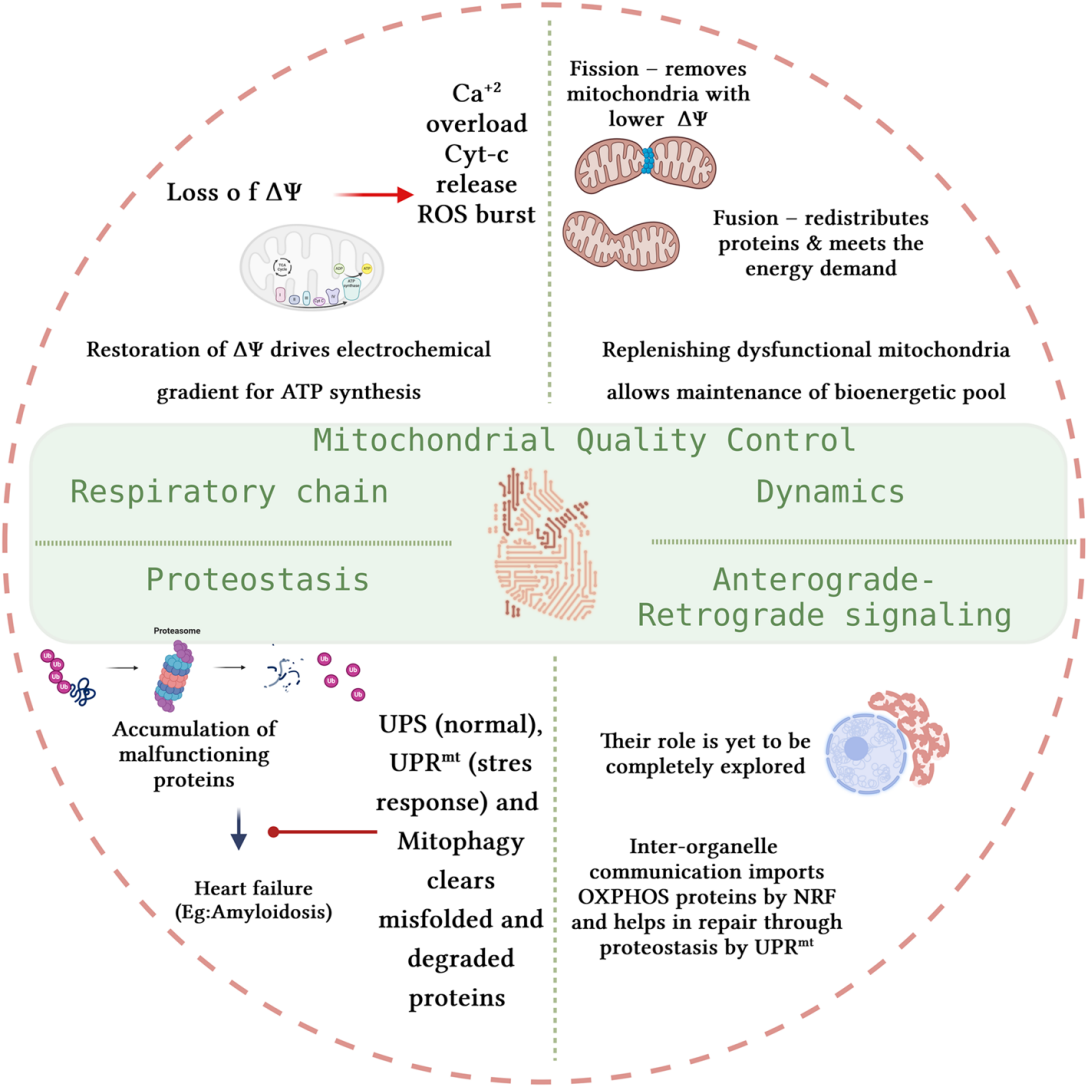
Mitochondrial contribution to cardiac homeostasis through quality control. ΔΨ-mitochondrial membrane potential, Cyt-c-Cytochrome-c, UPS-ubiquitin proteasome system, and UPR^mt^-mitochondrial unfolded protein response

While we have seen how mitochondrial dysfunction contributes to CVD risk, it is equally important to investigate the other side of the coin. Here we discuss how cardiac repair progresses following mitochondrial recovery and helps in remodeling to improve the heart’s function. A recent study indicated the responsiveness of dysfunctional mitochondria to drug treatment in diseased heart tissues that could treat cardiomyopathy in the future [121]. Several mechanisms of quality control are involved in cardiac repair and have been extensively reviewed by others [133]. We discuss here the perturbations that mitochondria undergo during the cardiac repair process and how they influence cardiac function as presented in Fig. 3. Mitochondria have been implicated as a metabolic driver of cardiovascular complications in induced and acquired CVD [134]. Hence the heart must maintain a fine balance of this organelle’s function to generate energy and limit ROS. Mitochondria have developed innate quality control mechanisms to sustain their function in times of imbalance to maintain the cardiac function through repair mechanism and homeostasis is imperative for mitochondrial function and cardiac health. Discussed further in the review is the role of the major contributors to quality control for mitochondria including ROS, proteostasis, dynamics, and antero-retrograde signaling.

### ROS

The DNA repair mechanisms in mitochondria are immature, which makes the mtDNA susceptible to damage by the oxidative stress caused by ROS. The ROS is a generated both as a consequence of normal ETC (Complex-I and III) activity or due to mitochondrial dysfunction, for example, monoamine oxidase in the heart [135]. Seminal studies by Hoppel and Lesnefsky group have significantly advanced our comprehension of ROS damage to mitochondria in the heart. These works demonstrate that inhibiting the ETC confers mitochondrial protection in ischemic hearts by preventing ROS-induced damage to cardiolipin and cytochrome-c [136, 137]. On the contrary, a balanced ROS level is necessary for signaling that promotes growth & proliferation as in the case of angiogenesis, contraction by stimulation of calcium channels, and regulates autophagy [138]. The antioxidant system (SOD, catalase, GPx, thioredoxin, and peroxiredoxin) in the mitochondria helps to balance this ROS and maintain physiological signaling and avoid undesirable effects. ROS scavenging has been a primary target to orchestrate cardiac repair followed by ROS-activated pathways such as the AMPK, and RAS and inhibition of ROS-producing enzymes. On the other hand, ROS promotes the transient opening of the mitochondrial permeability transition pore (mPTP) which is beneficial for heart function [139]. Multiple animal studies support the fact that reducing ROS generation through the modulation of the antioxidant system can enhance cardiac health and delay premature aging [140].

ROS is a double-edged sword, requiring tight regulation of its levels and posing a challenge for the proper administration of therapies. For instance, the antioxidant properties of H_2_S and its donors have proven cardioprotective benefits but the endogenous levels of H_2_S and functional mitochondria in heart determine the dose and a positive outcome for heart experiencing ischemia-reperfusion injury [141]. Hence, many mitochondria-targeted ROS scavengers such as MitoQ, SkQ, and SS31 more effectively protect the heart than the general ROS scavengers partly due to their effectiveness in preserving functional mitochondria and eliminating pathological ROS. Another important cardioprotective effect of ROS involving the mitochondria is provided by PKCε activation and translocation to mitochondria, induced by ROS. But a rather opposite effect is observed by PKCδ translocation, also triggered by ROS owing to the species involved which in this case was OH^*^ radical [142, 143]. Given that the ROS triggers several pathways, its cardioprotective effect depends on the functional mitochondria status.

The broad effects of ROS-mediated damage are obvious in endothelial dysfunction, hypertension, atherosclerosis, and HF. In particular, the involvement of mitochondria was noted by Lin et al. in patients with atrial fibrillation due to mtDNA damage leading to enhanced ROS [144]. Overall, we have a more complete understanding of the damage of ROS rather than its benefits. Studies of the benefits of ROS observed that ROS modulation regulated adipogenesis [145] in equine adipocytes, which is of potential use to prevent post-infarction remodeling of the heart where lipid deposition and fibrosis are the key reasons for HF. Gangwar et al., demonstrated the benefits of reactive oxygen and nitrogen species in the promotion of cell survival mediated by nitrosylation in murine cardiomyocytes subjected to hypoxia [146]. In a study by Long et al. (2020), muscle regeneration properties of flavonoid silibinin were attributed to increased ROS levels [147]. More recently, Li (2022) reviewed the impact of exercise on muscle health and proposed that exercise increases ROS and improves skeletal muscle activity by inducing epigenetic changes [148]. Despite these findings, the molecular mechanism behind protective ROS signaling remains elusive. It is also not clear if this signaling helps in the regeneration and recovery of damaged hearts and if so what is the role of mitochondria in the process.

### Dynamics in cardiac repair

The fusion/fission dynamics of mitochondria provide the organelle with much-required structural integrity in environments of stress [149]. It helps to maintain a healthy pool of mitochondria and therefore altered dynamics of the process are a cause of HF. Recent reviews by Lin et al. (2021) [150], Poznyak et al. (2022) [151], Quiles et al. (2022) [152], and Hausenloy group (2023) [153], extensively discuss how these opposing processes of fusion and fission of mitochondria are regulated through fusion proteins (MFN1,MFN2, OPA1) and fission proteins (DRP1, FIS1, MFF, MiD49/51). At present, we have a reasonable amount of evidence supporting that striking a balance between the two processes is key as low fusion or an excess of fission can trigger apoptosis. At a steady state, metabolic and energetic changes in the cell act as stimuli to promote the maintenance of energy production and are usually associated with morphological changes to mitochondria. The OPA1 protein has been recognized as a key regulator of this process by playing the dual role of being pro or anti-fusion [154, 155]. OPA1 is spliced into 2 forms by OMA1, the long unprocessed form (l-OPA1) and the short-processed form (s-OPA1). The ratio of these forms decides the fusion and pore opening of the inner membrane in the pro-fusion state. Enrichment of s-OPA1 was found to suppress fusion. In heart failure, reduced OPA1 levels have been reported from both human and animal heart tissues although the contribution of these forms in the heart is still unclear [156]. Supporting evidence shows that stress-induced depolarization of mitochondrial membrane due to ROS increases the activity of OMA1, which in turn converts l-OPA1 to s-OPA1, shifting the equilibrium to a pro-fission state [157, 158]. Besides fusion, OPA1 has pleiotropic effects of maintaining cristae morphology and respiratory efficiency, making it a sought-after drug target [159]. In 2018, ablation of OMA1 provided evidence of cardioprotection in three different mouse models of heart failure [158]. Developing OMA1 modulating drugs is a challenge given their off-target effects of cardiotoxicity which needs to be addressed [160]. The approach to promoting fusion or inhibiting fission for cardioprotection has taken center stage in recent times, especially with the development of a new class of drugs. These include small molecules such as M1 fusion promoter, and Mdivi1, a fission inhibitor that proved effective in several in vivo studies [161–164]. Additionally, the repurposing of known drug molecules that target mitochondrial dynamics is also being extensively explored for cardioprotection [165]. In particular, these approaches hold promise in diabetic cases where the underlying cause of heart failure is disengaged mitochondrial dynamics [164, 166]. Translation of these therapies to the clinic requires extensive evaluation of safety and efficacy in humans which is currently lacking for this new class of compounds. More details on such protective strategies have been recently reviewed by Hausenloy’s group (2023) [145]. However, as we found that mitochondrial subtypes are specifically prone to damage in various heart failure conditions such as IRI, finding the subtype affected and targeting them could help in faster recovery of the heart [167].

The present consensus is that improving mitochondrial fusion and/or reducing mitochondrial fission preserves the myocardium and several reviews have consolidated those findings [168, 169]. But the significance of this target strategy lies in the fact that this is well executed even in underlying pathological states such as diabetes [170]. Empirically, pathological remodeling is different from regeneration and stands as a barrier for stem cell applications to promote differentiation and proliferation in post-infarcted hearts. Mitochondrial dynamics holds as a promising target to promote regeneration as noted in vascular smooth muscle cells by antagonizing the GLP1 receptor [171]. On the other hand, GLP1 agonists have shown promising benefits in cardiovascular trials in diabetic patients [172] and hypertension [173], but whether they are effective in remodeling the same heart if challenged with infarction remains to be tested. A recent meta-analysis to date also supported GLP1Ra therapy to have only a moderate benefit in treating coronary lesions (MACE-Trial) [174].

### Proteostasis

Proteostasis is a process of regulated homeostasis of protein translation, folding, and degradation [175]. It involves numerous chaperones and regulatory proteins that are indispensable for the renewal of cellular proteins to maintain normal cardiac contractility. Normally, mitochondrial protein homeostasis is maintained by the folding and maturation of imported proteins, the guided refolding of misfolded proteins, and the degradation of non-assembled, damaged and misfolded proteins [176]. Interestingly, more than 40 independent proteases for mitochondria have been recognized that not only maintain proteostasis but also act as central regulators of organelle plasticity [177]. Chen et al., (2021) collectively reviewed the role of these mitoproteases in diseased hearts and emphasized that perturbations in this system precede mitochondrial dysfunction [178]. Dysfunctional proteostasis could be caused by oxidative modification of proteolytic proteins (LONP1) or mutation in controlling genes (*MIPEP*,* CLPP*,* HTRA2*) [176, 179, 180]. Rescuing these malfunctioning systems provides mitochondrial stability thereby protecting the heart from mitochondriopathies [179, 181, 182]. Overall, ‘mitochondrial proteostasis’ is controlled by the Ubiquitin proteasome system (UPS) via mitochondria-associated degradation (MAD), the unfolded protein response (UPR^mt^), and mitophagy. These systems have been extensively studied as therapeutic targets for diseases linked to mitochondrial quality control as recently reviewed by Hong et al. (2024) [183]. There is an extensive understanding of each of these systems as has been recently reviewed by others [184–189]. Although these systems work in tandem to remove unwanted proteins, the UPS primarily tags targeted proteins with ubiquitin and transports them to the cytosol for degradation while the UPR^mt^ is a stress response activated by the accumulation of unfolded/misfolded proteins within the mitochondria and degrades them via transcriptional activation of proteases through retrograde signaling [190].

The UPS has been implicated in several pathologic cardiac states such as hypertrophy, ischemia-reperfusion injury, and mitochondriopathies which have increased ubiquitin-positive deposits. Given its frequent role in HF, targeting specific proteases instead of upregulation of the entire system has shown a good therapeutic response [191]. Similarly, the enhancement of the UPR^mt^ response protects the heart from pathologic stress, making it a potentially valuable drug target [192]. On the contrary, while it is found that mitophagy markers are elevated in failing hearts, recent studies suggest that increasing mitophagy can alleviate microvascular endothelial dysfunction to prevent heart failure from ischemia/reperfusion injury [193, 194]. Miranda-Silva et al., studied this ambiguous role of mitophagy in remodeling but could not explain the elevation in mitophagy found both during the presence (overload) and absence (unload) of stress [195]. It is postulated to be an important quality control action to maintain mitochondrial bioenergetics. Hence it is not clear if mitophagy is beneficial or harmful for cardiac remodeling and is a subject of research focus. Given current knowledge of the role of proteostasis in heart failure, future studies may wish to focus on how these systems are interconnected with an emphasis on the time course of drug intervention and monitoring mitochondrial quality.

### Nuclear-mitochondrial crosstalk

The topic of nuclear-mitochondrial crosstalk merits an entire review to itself and therefore this section only briefly highlights studies relevant to cardiac function. The OXPHOS machinery has only 13 proteins encoded by the mtDNA while the rest of the proteins (1,140 mouse) are imported from the cytosol [196]. The translocation is necessary to produce functional mitochondria to meet the energy demands of the cell and suggests the need for a well-orchestrated signaling mechanism from both ends to meet the working demands of the myocardium. This bidirectional signaling; anterograde (nucleus to mitochondria) and retrograde (mitochondria to nucleus) is critical for cell-plasticity but not comprehensively explored as to how they maintain the cardiac physiology and contribute to pathological states [2]. Evolving studies in the field of mitochondrial research such as the evidence of mitochondrial splicing variants generated by nuclei-encoded spliceosome complexes suggest that there is an ocean of information hidden in the signaling process yet to be discovered [75]. One such emerging case study reported nuclear control of mtDNA replication by topoisomerase (TOP3A) and its involvement in dilated cardiomyopathy [197]. The study observed multiple mtDNA deletions with predominant myalgia and weakness suggesting energy deficiency disorder, but further studies on TOP3A’s function and pathological role are yet to be established.

Analogous to the above case study, a well-studied nuclear-driven signal that controls mitochondria is the energy shift from glucose/lactate in the prenatal hypoxic environment to fatty acids during postnatal conditions, driven by the metabolic sensor Hypoxia-inducible factor (HIF), whose discovery was awarded the Nobel Prize in 2019 [198, 199]. Regarded as the master regulator for oxygen homeostasis, HIF helps muscles generate ATP from lactate during stress, reducing its dependence on OXPHOS. Additionally, HIF upregulates SOD2 to suppress ROS in mitochondria. Semenza (2014) has comprehensively reviewed HIF’s role in cardioprotection, part of which is through metabolic reprogramming that helps recovery of heart following ischemic injury [200]. Following prolonged hypoxia/ischemia, the perinuclear mitochondrial distributes and activates the HIF leading to cardiomyopathy in the long term due to chronic expression impairing heart function. Thus, HIF-stabilization has been a key strategy for promoting contraction and reduction of infarction [201, 202]. Factually, HIF induction is controlled by mitochondria as depletion of mitochondria DNA in ρ^0^cells or inhibition of ETC function prevents degradation of HIF [203]. Recent evidence also suggests that mitochondria-targeted HIF protects the liver from oxidative stress by downregulating mtDNA-encoded mRNA without affecting the organelle content emphasizing its non-transcriptional role under hypoxia [204]. However such roles with relation to HIF or its mitolocalizing isoforms (HIF3α) or such signaling mechanisms in general are not fully understood in cardiovascular disease [204].

The endosymbiotic origins of mitochondria in eukaryotic cells have repositioned its genome to integrate with the host’s nucleus [205]. Most of the nuclear-mitochondrial crosstalk is ‘anterograde’(95%), that is, nuclear transcripts influencing mitochondrial gene expression, which is evident from the presence N-terminal mitochondrial targeting sequences (NUMTs) present in protein-precursors imported to the mitochondria; garnering significant research attention [206]. Thus, it would be an understatement to say that mitochondria depend on the nucleus for their existence. Prominently, the anterograde signaling pathways promote mitochondrial health by activation of genes encoding mitochondrial proteins through transcriptions factors such as NRF1&2. In response to energy status (level of ATP/ADP, NAD/NADH, TCA cycle enzymes and membrane potential), the activation of nuclear receptors (PPAR delta and EER) driven by PGC1α promotes the genes required for regulation of TCA cycle and ETC-proteins. Such regulation is mediated by signaling involving the AMPK, AKT and SIRTUIN pathways. For instance, AMPK activation promotes biogenesis, and increases membrane potential and fatty acid oxidation, thereby providing energy for cardiac contractile function, and differentiation as demonstrated in a recent work on human induced PSC derived cardiomyocytes [43].

Unlike the role of retrograde signaling in the developing heart, the role of it in pathologically challenged hearts is poorly described. Two major pathways identified in retrograde response are the RTG (retrograde) and TOR pathways studied extensively in yeast systems and the lack of mammalian homologs of the proteins in the pathway has left us with unvalidated results [207]. It is known that mitochondrial stress initiates the translocation of transcription factors such as SIRT, RTG and FOXO that drive the transcription of genes that promote antioxidant response or apoptosis dependent on the signal. Even the molecules released from the mitochondria (mainly the Ca^2+^, ROS and protein breakdown products of UPR^mt^ response) that affect nuclear gene transcription are accepted as canonical retrograde signals [208]. One such gatekeeping retrograde signal (ATFS-1) protected *C.elegans* from anoxic stress [209]. Under proteolytic stress conditions, ATFS-1 import to the mitochondria is blocked and it accumulates in the nucleus to activate the target genes. Emerging evidence suggests that its mammalian ortholog family of genes; Activating transcription factors (ATF) play an important role in cardioprotection as well [210–213]. The stimulus for activation is thought to come from protein misfolding response (UPR^mt^), which has been identified as an emerging drug target for cardioprotection [213, 214]. The immunostimulatory role of mtDNA is also one of the factors in eliciting retrograde response. Especially, a supercoiled confirmation of mtDNA called the Z-DNA is reported to activate the ZBP1 expression and sustain an inflammatory response that contributes to Doxorubicin-induced cardiotoxicity [215]. ZBP1 is a key innate immune regulator, whose role in CVD pathologies is yet to be explored. More recently, mitochondria-derived peptides (humanin and MOTS-c) were discovered to be involved in CVD pathologies [216]. However, their targets and signaling mechanisms must be studied before they can be harnessed for treatment [217].

Despite knowing these signals, identifying the networks and responses in the cardiac physiology is still a challenge that needs to be worked on [2]. New methods of single-cell sequencing and GWAS studies can help to understand these pathways in in-silico systems and can be validated in working models. Likewise, the somatic mutations and the post-translational modifications that affect the signaling and its interaction are poorly understood (Ex: OPA1). There is now a demand for animal studies focusing on nuclear-mitochondria crosstalk to identify individual populations at risk, drugs that interact with signaling (toxicogenomics) and identify new targets (pharmacogenomic).

### The energy polarity in maintaining cardiac function

The heart is dynamically able to derive energy from numerous metabolic sources (e.g., fatty acids, carbohydrates, BCAA, and ketone bodies). These substrates compete for the OXPHOS at the mitochondria by providing acetyl-CoA [218]. Despite availability of all the substrates, ATP production is contributed to by the oxidation of fatty acids and carbohydrates (90%). Under normal physiological conditions, BCAA and ketone bodies are known to have less (10%) contribution to the overall ATP production. Since most CVD complications arise in patients with metabolic alterations as a temporal phase transition, it is important to analyze the influence of increased levels of specific intermediary fuels on cardiac function and progression of disease. Also, groups have reported that targeting mitochondrial dynamics and proteolytic pathways seem to improve cardiac function by metabolic remodeling even in disease states but without a clear consensus of the process to derive energy for remodeling [170, 219]. This emphasizes the need for basic research to explore the energy polarity for various substrates and their relation to cardiac function, signaling, genetic influences, and recovery from pathological states.

Cardiac energy substrates clearly have a metabolic relationship to one another and also function as signaling molecules influencing the mitochondria. Muoio and Neufer (2012) proposed that the nutrient induced mitochondrial stress caused by accumulation of acetyl CoA fosters hyperacetylation leading to ROS production, decrease in OXPHOS capacity, impairment of glucose uptake and insulin sensitivity [220]. Similarly, ketone bodies inhibit HDAC protein to control gene expression, and in pathological states, the heart relies on ketone metabolism to derive energy [220–222]. The mechanistic link between the energy substrates, their metabolism and contractile function remains obscure and establishing them could be of potential benefit for metabolic therapy (recovery) of failing hearts [223].

Aubert et.al’s (2016) quantitative proteomics of heart failure in a mouse model showed downregulated fatty acid metabolism and elevated ketone oxidation in mouse hearts [222]. Recently several studies have recognized ketone therapy as a clinical opportunity to treat heart failure giving a new direction to the heart as a ‘metabolic omnivore’ [224]. Opposing views have been proposed by Brahma (2022) stating that ketones are not crucial for the maintenance of normal homeostasis and are utilized only in the presence of heart failure [225]. Likewise, catabolic defect in BCAA was noted as a feature of heart failure [226] and the clearance of accumulated intermediates recovered the cardiac function. Benefits could be derived by simultaneously targeting the mitochondria as well since the elevated BCAA suppressed OXPHOS and increased ROS in the isolated mitochondria from the same study.

#### Genetic regulation of energy utilization in heart

Under pathological conditions, short-term energy substrate regulation is based on demand/supply, controlled by signaling mechanisms induced by biomolecules such as insulin and glucose. But in long-term conditions like hypertension, atherosclerosis, and diabetes, the induced changes in metabolism are regulated transcriptionally. The major transcriptional regulator in such cases is the HIF family of proteins, the activation of which promotes glycolysis and suppresses fatty acid (FA) oxidation. Liu (2020) found that suppressed myocardial HIF-1 in cyanotic congenital heart disease resulted in higher levels of glycolytic intermediates, and enhanced substrate utilization by the mitochondria for glucose instead of FA [227]. HIF also regulates cardiac lipid metabolism as elevated HIF family genes contribute to atherosclerosis [228], hypertrophy and remodeling [229, 230]. In the case of type-2 diabetes mellitus (T2DM), abnormal metabolic phenotype of HIF-1α destabilization is noted as less tolerance to hypoxia and rapid HF progression and its stabilization by Molidustat was effective in the treatment of diabetic heart from post-ischemic recovery [231]. Several such specific nuclear receptor transcription factors and co-activators regulate the genes and control the metabolic substrate preference which is yet to be explored. The significance of the shift in substrates for ATP synthesis in failing heart can also trigger cardioprotection as observed in hormetic strategies adopted to reduce myocardial IRI such as ischemic preconditioning, postconditioning, remote ischemic conditioning either by transient activation of signals or genetic changes. Such cardioprotective strategies are known to offer a second window of protection, which is far from being completely understood [232, 233].

**Fig. 4:**
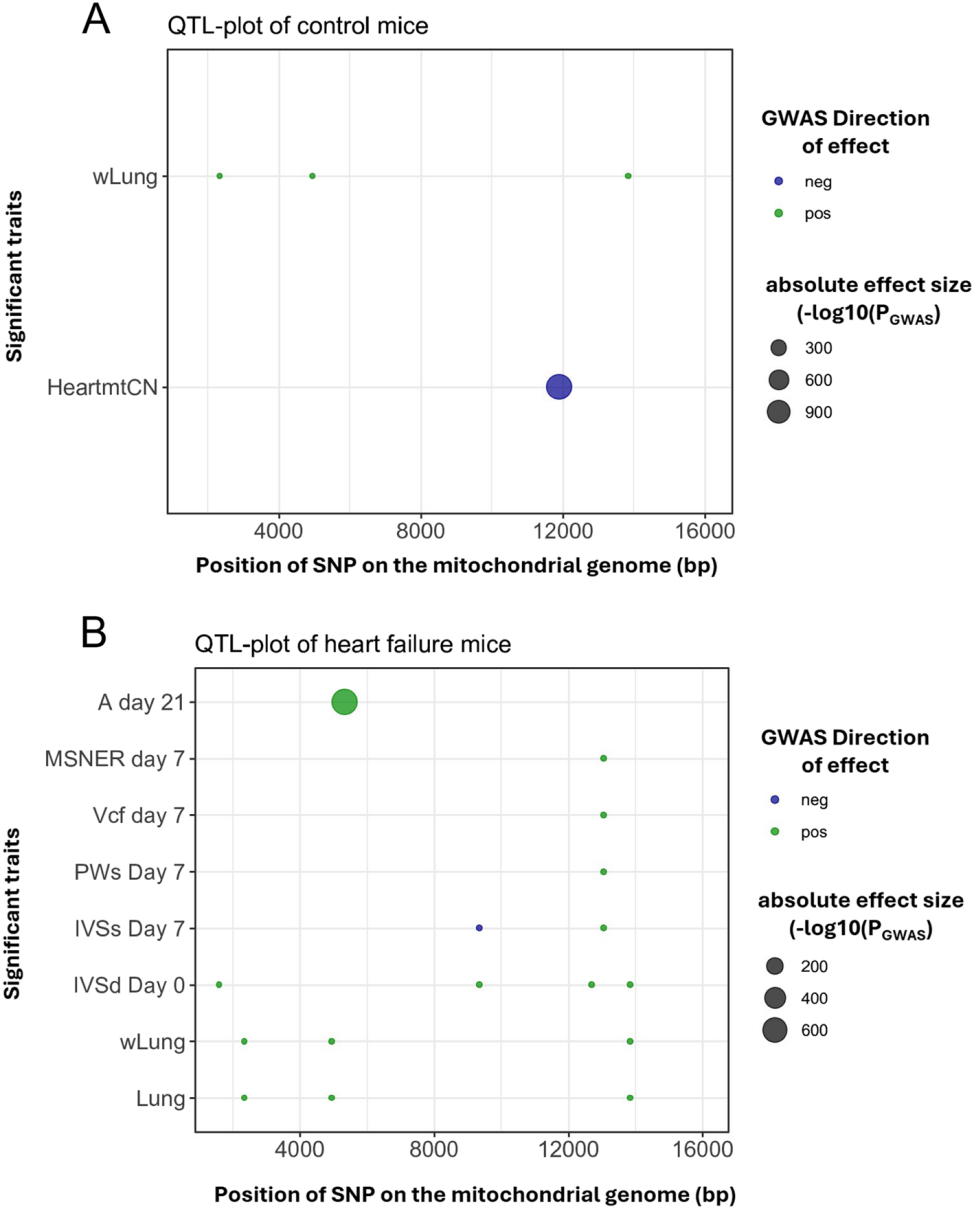
QTL map associating mtSNPs at significant (LOD > 3) loci with cardiac traits in HMDP mice from (**A**) control and (**B**) isoproterenol challenged groups. Lung-Lung weight, mtCN- mitochondrial copy number, A-Late mitral inflow velocity-, MSNER-Mean normalized systolic ejection rate, Vcf- velocity of circumferential fiber shortening, PWs- posterior wall thickness at end systole, IVSs- interventricular septal wall thickness at systole, IVSd- interventricular septal wall thickness at diastole, and wLung- normalized lung weight

**Fig. 5:**
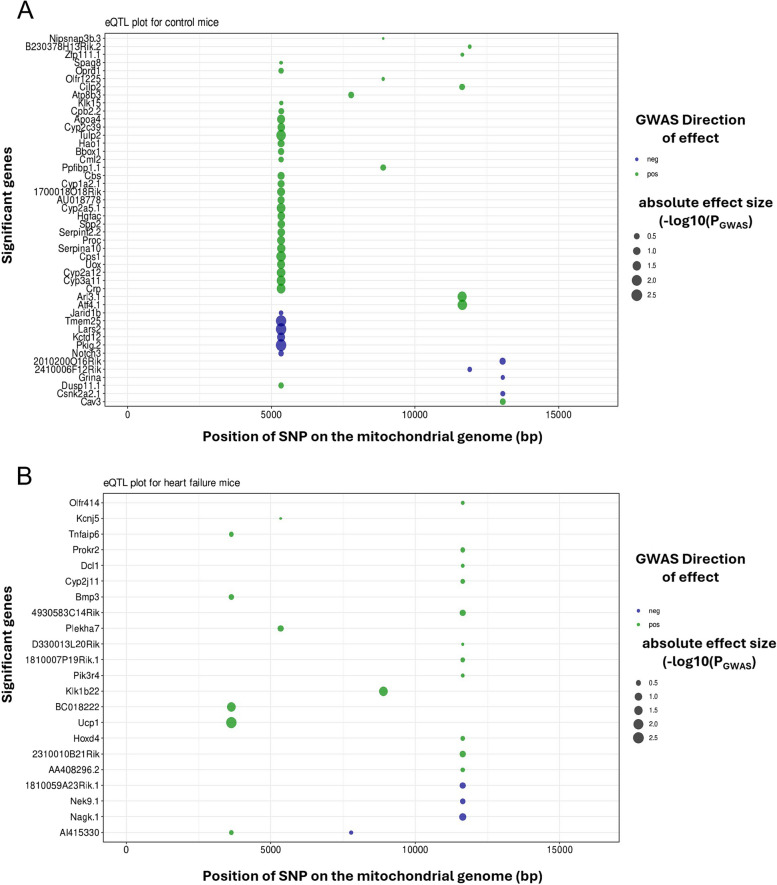
eQTL Plot of significant (LOD-threshold > 10) gene interactions with mtSNPs of HMDP mice strains from (**A**) control and (**B**) isoproterenol-challenged groups

 Studying the crosstalk between metabolic substrates and cardiac phenotype in heart failure is limited by the fact that large number of factors and signaling influences play a role in controlling the cardiac metabolism and function. Such situations could be managed by the use of computational tools (GWAS, and Network analysis) to find the role played by the energy substrates in maintaining the cardiac function. For instance, using systems genetics approaches, we previously published GWAS for heart failure in the hybrid mouse diversity panel (HMDP), a genetic reference population consisting of a large number of inbred mouse strains [234]. The study identified previously unrecognized genetic variants on the nuclear genome that contributed to heart failure-associated phenotype in a large panel of genetically diverse inbred mice. When we examined the mitochondrial single nucleotide variants (mtSNV) associated with the mouse strains from the HMDP for similar phenotypic links (Figs. 4 and 5), we found a significant association of several physiological parameters with isoproterenol-induced HF. Interestingly, heart mtDNA copy number appears to be negatively associated with the diversity in these mice [235] which supports studies in the field that associate reduction in mtDNA copy-number as a risk factor for HF [125, 127, 235, 236]. Further studies to characterize various mitochondria-related phenotypes as discussed in Sect. 2 and establishing a clear role for the mtDNA SNPs across the subtypes in CVD will unfold new signaling mechanisms.

### Metabolic shift as a compensatory mechanism

ATP is an essential energy source for heart function. The imbalance in ATP generation/utilization due to heart failure is of great concern. In the heart, the majority of ATP is generated by fatty acid metabolism rather than glucose metabolism but at the expenditure of high oxygen consumption [237]. This preferential dependence of heart, liver and muscles towards fatty acids can undergo a shift in limited oxygen conditions, switching to glucose oxidation. How efficient these two processes are in sustaining the contractile function of the heart remains a topic of debate. Additionally, ketones and BCAAs also can be consumed to produce energy for the heart in processes that are often seen in HF [218]. Despite several substrates present, most of the processes end up in the mitochondria for ATP synthesis and calcium uptake, which is why it is essential to connect mitochondrial (organelle) function to the mechanical functions of the heart. At this point, it is unclear whether ATP depletion is the reason for HF because it limits the hemodynamic function of the heart, often termed the energy starvation hypothesis [238] or whether disease progression leads to metabolic dysregulation of energy generating processes. In a recent study by Abdalla (2023), evidence indicates that complex epigenetic regulation drives ATP depletion at a very early stage even before the loss of cardiac function, and is associated with the downregulation of genes encoding the proteins for mitochondrial NAD^+^ synthesis pathway [239]. Translational advantage of NAD^+^ supplementation to restore mitochondrial respiration in hypertrophic hearts was demonstrated in human hypertrophic cardiomyopathy by Nollet (2023), clearly indicating the cause and the solution [121]. Nollet’s group observed that in patients with obstructive hypertrophic cardiomyopathy, improper arrangement of mitochondria was strongly associated with dysfunctional mitochondrial respiration. Restoration of NADH levels by supplementation with NAD^+^ restored mitochondrial respiration and could be a valuable treatment option [240].

ATP production and utilization are highly regulated processes and its yield from substrates is in the order of FA(106) > BCAA(40) > glucose(32) > ketones(22). Though BCAA generates a substantial amount of ATP, its contribution is < 5% in the heart under normal circumstances as FA oxidation meets the ATP demand. In conditions of high demand, ATP production is supplemented by glycolysis and the phosphotransferase reactions [241]. However, chronic high demand, as observed in CVD, activates the sensors controlling metabolic remodelling leading to reduced utilization of ATP along with impairment in its production by any of these routes (fatty acid, glycolysis, BCAA, ketones, creatine kinase, and adenylate kinase). Thus, rather than simply regulating ATP synthesis/degradation, the pathways that control its production/utilization play a key role in cardiac pathology as explained by Ingwall [238, 241]. In fact, impaired activation of mitochondrial Potassium channels appears to be the reason for impaired responsiveness to conditioning strategies/drugs, especially in aged hearts [242]. These reports force us to rethink the actions of various drugs to study their role in ATP generation or their effects on the processes/pathways/channels that require ATP. As demonstrated by Molidustat and NAD^+^ administration, modulation of metabolic demand can act as a powerful treatment against HF progression by restoring mitochondrial function [121, 231]. In conclusion, it is essential to assess the metabolic demand of the heart, and its function based on substrates and effectively design strategies for recovery from HF.

## Canonical pathways of cardioprotection via mitochondrial signaling

### Pathways

From our earlier discussions, we have sufficient evidence indicating that targeting the mitochondria can rescue the failing heart as most of the disease-implicated signaling pathways converge at mitochondrial organelle function. Table 3 summarizes the list of cardiac-involved pathways and the changes that disruption of these pathways brings about in the organelle and its function. The outcome of disrupted signaling is an altered cell metabolism and/or an inhibition of apoptosis. Additional emphasis should be placed on the presence of functional mitochondria for effective cardioprotection, the absence of which could significantly derail the efforts of targeted therapies [141].

**Table 3:** Mitochondrial convergence of cardioprotective pathways

S.No.	Major Pathway	Key downstream molecule affecting mitochondria	Interaction with mitochondrial component	Phenotype changes in mitochondria due to activation of cardioprotective pathways	Ref
1	RISK (PI3K/Akt, MEK/ERK)	GSK3β, PIM1, cGMP/PKG	mPTP	Closing of mPTP prevents swelling and rupture of mitochondria	[243]
2	SAFE (JAK-STAT3)	STAT3, GSK3β	mPTP and complex-I	Opening of mitoKATP channels causes depolarization and reduces Ca^2+^ uptake to maintain the matrix volume	[244]
3	NO/PKG-PKC pathway	GSK3β, NO, PKG and PKC	PKG and PKC with mPTP and mitoK_ATP_ channels, NO is inhibitor of cyt-c oxidase activity and respiration competitively to O2, S-nitrosation of proteins	NO causes increased mitochondrial membrane potential and activation of cytoprotective signaling	[245–247]
4	PI3K/Akt/mTORC1 (Insulin mediated)	mTORC1/MCL-1	ATP citrate lyase, MCL-1	-	[248, 249]
5	Adenosinergic (Activation of A1 and A_2A_ AR)	Multiple effectors such as Protein kinases, HSPs and HIF1α	mPTP and mitoK_ATP_ Channels	-	[250]
6	Mitophagy and Ubiquitin-Proteasome	PINK1 (membrane depolarization sensor), PARKIN, MFN2 and Ubiquitination of adaptor proteins (p62/SQSTM1 and Optineurin) Short lived mis-folded mutant proteins	PINK1 functions beyond mitophagy to control mitochondrial functions by regulating complex-I activity in response to stress while Deubiquitinases play key role as recruitment, activation, import and export of proteins to the mitochondria	Activation maintains mitochondrial integrity and stabilizes membrane potential apart from promoting mitophagy	[251–253]
7	PKCε	PKCε phosphorylates Cx43	PKCε interacts with VDAC, ANT, and HKII to control the permeability pore transition. It Also activates mK_ATP_ channels	Increased mitochondrial swelling is prevented by inhibition of PKCε	[254]
8	Mito Connexin 43	Cx43 is a component of IMM only in SSM	Regulates K + fluxes via mitoK_ATP_ channels	The sites of phosphorylation determine the phenotype and is yet to be explored	[255, 256]
9	Autocoid stimulation-GPCR (Adenosine, Bradykinin, Opioids)	PKCε,NO, STAT3 and GSK3β.	Cx43, mPTP and mitoK_ATP_	Connexin reduction is associated with reduced K^+^ influx and swelling. It lowers ROS and reduces cardiac infarct size	[257, 258]
10	MOSPD contact site with ER- novel area of search	NA	NA	NA	[259]
11	Uncoupling proteins	ROS	UCP3, 2 and 1	Transporters that regulate discharge of proton gradient and acts as calcium regulator to maintain thermogenesis and structure	[260, 261]

*RISK* reperfusion induced salvage kinase pathway, *SAFE* survivor activating factor enhancement pathway, *mPTP* mitochondrial permeability transition pore, *mitoK*_*ATP*_ ATP activated mitochondrial potassium channels, *PKC* protein kinase C, *PKG* protein kinase G, *VDAC* voltage-dependent anion channel, *ANT* adenine nucleotide translocator, *UCP* uncoupling protein, *CX43* connexin 43, *GSK* glycogen synthase kinase, *mTOR* mammalian target of rapamycin, *NA* not available and yet to be explored

### Differential impact

A larger puzzle that remains to be solved is the differential impact of signaling on the interfibrillar (IFM), subsarcolemmal (SSM), perinuclear (PNM) and intranuclear (INM) mitochondrial subpopulations present in the heart. The seminal investigations led by Palmer, Hoppel, and Tandler in the 1970s–80s demonstrated that IFM had superior substrate oxidation capacity and better calcium handling compared to SSM [262, 263]. These adaptations of mitochondrial subtypes are critical in pathological conditions as they shape the recovery and remodeling of the heart. However, such observations agree to differ with the animal model and pathology as best described in a recent work by Shekar (2022), demonstrating that IFM and SSM in porcine hearts subjected to AMI underwent the same significant dysfunction without any difference in calcium handling or ATP production [264]. According to the group, oxygen consumption was the biggest difference among the subtypes. At this juncture, we need to rethink if the sub-type differences help in remodeling especially using more clinically relevant large animal models. Another key aspect of mitochondrial subtype differences is their response to drugs which has been demonstrated to impact the recovery from ischemia-reperfusion injury [167, 265, 266]. Studying mitochondrial toxicity in drug discovery is a different aspect dealt with elsewhere [267]. Besides the obvious spatial and functional differences among these mitochondrial sub-populations, exceptionally little information is available as to how these subtypes behave differently in the presence of acute and chronic stress [268, 269]. For instance, the pioneering study by Hoppel (1982) provided evidence of selective involvement of IFM in cardiomyopathy [270]. Subsequent studies in diabetic hearts also showed declined respiratory activity in IFM but not in SSM [113]. Such differences also impact the protective strategies and signaling that otherwise work in normal conditions [129] and additionally negate the cardioprotective effect of previously established drugs [271]. Interestingly, a recent study by Rajab (2022) found structural alterations in mitochondria induced by early diabetes in mice [272]. The recent data from the Antentor Hinton lab suggests that the mitochondria undergo various morphological changes due to aging and can potentially link to HF phenotypes observed in humans [273]. A major drawback in the field is that most of this information is available for only the most widely studied subpopulations: IFM and SSM due to their relative abundance and established isolation protocols. Even these relatively more studied subpopulations suffer from a lack of research interest: the last paper to address exclusive differences between IFM and SSM subtypes in terms of markers expressed was published in 2009, in which it reported that the presence of connexin43 is localized in SSM but not IFM [274]. In a recent thematic issue in the Philosophical Transactions of the Royal Society, Voglhuber (2022) compared for the first time IFM and PNM in heart failure [275]. Their study reported the role of PNM in shaping nucleoplasmic calcium levels thereby controlling the cell microenvironment and hypertrophy progression. Advancements in single-cell omics platforms have provided more insights into subpopulations of cardiomyocytes in hypertrophy, providing more challenges for researchers to explore, leaving behind clues for investigating the mitochondrial subpopulations in a similar manner [276]. Together they may form the deciding factors in shaping the cardiac phenotypes.

### Genetic regulation

Somatic mutations in mtDNA are increasingly being recognized as contributors of cardiovascular disease but our understanding of its role is restricted by the lack of mechanisms that explain the outcomes due to heteroplasmy and the threshold effect [134, 277, 278]. Age and haplogroups remain a major contributor of these mutations apart from underlying conditions such as diabetes, hypercholesteremia, and hypertension which are typically associated with CVD cases [279]. Though it has been recognized that most mtDNA mutations are present in the D-loop region and the 12 S rRNA, a recent report by Calabrese (2022) has recognized no specific mutational hotspots exist for these widely distributed mtSNV across stroke, hypertension, or ischemic heart disease [279]. But these mutations are, however, strongly associated with age and could possibly be a result of genomic mutations caused in the mitochondrial regulatory genes such as TFAM and POLG [280]. In 2019, McManus demonstrated for the first time the interaction between mtDNA and nDNA augmenting the progression of cardiomyopathy in C57BL/6JEiJ mice [281] and was later elaborated by Lim (2021) in humans [282]. Lim identified 11 different genotype-specific cardiac involvements in patients with mitochondrial mutations suggesting a diagnostic use for the mtDNA mutations. This rapidly evolving research in the field could be translated to new therapies in development by harvesting the benefits of single cell/nucleus/mitochondrial profiling and gene editing for treating CVD. For instance, using our data of nuclear gene expression in HMDP, we constructed an eQTL (Expression quantitative trait loci (eQTL) – statistical method linking phenotypic data (traits/gene expression) and genotypic data (molecular markers/SNPs) [283]) plot explaining the interaction of mtSNPs (mitochondrial Single nucleotide polymorphisms) with differential gene expression in HF induced by isoproterenol (Figs. 4 and 5). The eQTL suggested a significant number of genes involved to be associated with loci in the mtDNA, whose expressions were affected by the mtSNPs in the HMDP from both control and heart failure groups. In the coming sections, we provide insights into how newer computational approaches provide useful information on the regulation and function of mitochondria and their relationship with disease progression.

## Computational approaches provide insights into mitochondrial function and disease

Over the past two decades, computational and ‘omics-scale approaches have revolutionized our ability to understand the heart [284]. For example, gene expression analysis has progressed from traditional qPCR to microarrays before transitioning to the era of next-generation sequencing with bulk and now single-cell and spatial transcriptomics sequencing approaches. Additionally, advances in genotyping, DNA sequencing, and GWAS approaches have revealed new insights into the relationships between polymorphisms and disease. Taken together, these new approaches provide new insights into CVDs, mitochondrial anomalies, and overall physiological and pathological states in the heart [285, 286]. Concomitantly, mutations in mtDNA are associated with CVDs and can originate from maternal inheritance, epigenetic modification, environmental and lifestyle factors as discussed in detail elsewhere [287]. In this section, we describe some recent advancements in the study of mitochondria in the heart using systems genetics.

### Bulk RNAseq and mitochondria

In mitochondria-rich cardiomyocytes, up to 80% of all sequencing reads are soaked up by mitochondrial genes, yet an early filtering step in most RNAseq protocols is the removal of all mtDNA-associated gene expression [288]. This results in a loss of significant amounts of useful data and leaves the researcher with only ~ 20% of their original reads for downstream analysis especially in tissue like heart which comprises of highest percentage of mtDNA [289]. It is likely that a closer examination of this ‘junk’ RNA would reveal powerful indicators, mediators, and drivers of CVD. For example, mitochondrial dysfunction predisposed murine hearts to RV failure in a study by Hwang (2021) as it turned out to that the most significantly affected pathway has genes related to ETC [290]. Similarly, increased non-pathologic heteroplasmy in a mouse model predisposed them to metabolic stress in adulthood leading to not only heart failure but also affected other organs, causing premature death [60].Although, the hearts were sequenced on a Illumina HiSeq platform, the authors missed out on the mtDNA sequencing which could have given more critical information on mRNA abundances, long non-coding genes, mRNA information on mitochondria encoded peptides, as elucidated by Dunin (2019) in fish hearts [291]. Another advantage offered by mtRNA sequencing is that the heteroplasmy is transcribed with < 5% difference from mtDNA [292] which could be used to explain the cardiac phenotypes. Bulk RNAseq, however, has several limitations that other methods, such as sc/sn-RNAseq or long-read sequencing are able to address more clearly. For one, bulk RNAseq struggles to differentiate Nuclear-Mitochondrial DNA segments (NUMT) from genuine mtDNA transcripts which can significantly reduce the efficacy of the method. Furthermore, bulk RNAseq reports results on a heterogenous population of cell types, locations, and temporal profiles, whereas other approaches such as snSeq/scSeq or spatial transcriptomics combined with computational approaches avoids these issues by focusing on mitochondrial dynamics at the individual cell level.

### Single Cell/Nucleus-RNAseq and mitochondria

Single Cell approaches have exploded in popularity over the past few years as a means of understanding the underlying role of individual cells in physiology and disease. Limitations in the most popular single cell sequencing (scSeq) platforms (e.g. Fluidigm or 10x Chromium) prevent researchers from studying cardiomyocytes specifically due to their size and asymmetrical shape [293], however there is robust data generated at the nuclear level and exciting new developments [294, 295] that suggest that cell-level analyses will soon be possible in the heart. Despite these challenges, sc/snSeq approaches have proven specifically useful in understanding the heterogenous cellular populations of the heart, providing researchers with enormous amounts of data compiled in Tabula Muris [296], human heart [297] and the ENCODE project [298] on transcriptomic heterogeneity not only in normal but also in diseased hearts across multiple distinct cell populations. When combined with new advances in spatial transcriptomic mapping [299], reports suggest that we may be at the start of a new generation of precision medicine in which drug responses are understood at an individual level by analyzing patient-specific cellular heterogeneity in disease states. Although such an approach has many hurdles to pass before it is able to be implemented in the clinic [300], the big-data generated in these approaches helps to pave the way for integration of studies to identify disease transcriptional patterns [301] and establishing comprehensive cell-atlases for the heart [297, 302]. With rapid advancements in the field of scRNAseq, causal links of cellular lineages towards experimental myocardial infarction have been identified [301]. Despite these advances, most of the above referenced studies totally ignore the data captured from the mitochondria due to faulty assumptions regarding data cleaning owing to maintaining the quality of analysis, heteroplasmic nature of the mtDNA and experimental necessities of using nuclei rather than whole cell. Given the importance of transcriptomic anterograde and retrograde signaling from nuclear and mtDNA respectively, it is necessary the organelle be not neglected.

In 2018, Nomura et al. found transcriptional increases in mitochondrial genes associated with oxidative phosphorylation and biogenesis due to pressure overload in mice. They mapped single cardiomyocyte transcriptomes by reconstructing the trajectory of signatures that lead to hypertrophy and showed an association of increased mitochondrial biogenesis with cardiac hypertrophy as a compensatory mechanism for high energy demand [303]. A direct evidence of heteroplasmy affecting metabolic functions of cells and causing multiple comorbidities including heart failure has been reported by the José Antonio Enríquez’s group in a well-characterized mouse model [60]. Cell-type specific gene regulatory networks were also noted in snRNA-seq studies on postnatal hearts which served as a rich source of data sets for analysis of mitochondrial cardiomyopathies [304]. Additional studies using these previously overlooked sources of mitochondrial information are crucial to further advance our understanding of cardiac signaling and the relationship between this crucial organelle and organismal homeostasis and survival.

### Long Read Sequencing and the mitochondria

Despite the successes of many sequencing approaches to studying the role of polymorphisms and structural variations that underly the phenotypic diversity of the heart in both stressed and unstressed conditions, there are key downsides to the commonly used short-read paradigm that are being addressed by the advent of new long-read sequencing approaches such as the nanopore by Oxford Nanopore Technologies, single-molecule real-time sequencing by PacBio, and the synthetic technologies adopted by Element Biosciences, MGI, Illumina and 10XGenomics [305, 306]. One key flaw of short-read sequences is the inability to distinguish between large-scale duplications of DNA sequences. This is of key importance for mitochondria as NUMTs are broadly misidentified as mitochondrial sequences when they are, in fact, nuclear in origin.

Long read sequencing relies on microfluidics and light or electric current-based technology to generate significantly longer reads than are possible using the standard synthesis and ligation-based approaches. Long Read sequences are easily able to reach 16Kb size of mitochondrial genome in less than 2 h and offer a unique means by which researchers can fully understand the entire structure and sequence of the mtDNA across its sub-cellular populations. For example: Pollet (2020) provided a benchmark mtDNA variant identification in equines which could be applied to other animal species [307] and Vandiver’s data establishes the relation between mtDNA deletion and aging using this rapidly rising technology [308]. This is an exciting field of active research that should be more fully developed for cardiovascular research in the coming years for the detection of rare mitochondrial genetic variants across the mitochondrial subtypes.

### Resources and repositories for mtDNA studies

In contrast to the large number of repositories for other forms of genetic/transcriptomic/phenotypic data, there exist limited working online repositories for mammalian mtDNA-associated research. Currently, human mitochondrial genome research benefits from MITOMAP (https://mitomap.org/foswiki/bin/view/MITOMAP/WebHome), MSeqDR (https://mseqdr.org/mitobox.php), and HmtDB (https://www.hmtdb.uniba.it/about) which host the database and tools for analyses of human mtDNA variations as reviewed by Cappa [309]. Murine data is nonexistent on these platforms and is difficult to acquire through other means. For example, the two major resources MitoCarta and MioBreak are limited to an inventory of mouse genes encoding proteins that localize to the mitochondria and mtDNA break points. Researchers exploring the NIH’s sequence read archive (SRA) and gene expression omnibus (GEO) find it difficult to specifically obtain data on the mitochondrial genome as it is not a prioritized feature in these datasets. Hence, a well-curated database of studies exploring the mitochondrial genome in non-human species under various pathological conditions is required. There are, however, some growing resources for analyzing mtDNA sequences once they are obtained such as the MitoMiner 4.0 [310], MitoZ 3.5 [311], MitoSeek [312], MitoFinder [313], BamSignals [314], MseqDR [315], and Mitohelper [316]. These tools help in implementing wide range of functions such as data mining, annotation, heteroplasmy and mutation detection, and visualization either as a standalone function or integrating into an environment of graphical step by step usage such as Galaxy [317]or mitoXplorer [318]. A major caveat in these resources is the lack of curation, species specificity, inter-platform integration and user-restricted access. With the increasing advent of sequencing technologies, the above-discussed data will become common and require resolute, streamlined storage and analysis platforms.

### Aggregation of data points into predictive models in the mitochondria

Due to the low effect sizes of many individual GWAS loci, there has been an evolving trend in the field to instead develop polygenic risk scores (PRS),;measures that integrate large amounts of information about individual polymorphisms into a single predictive measurement. This score estimates the genetic liability for an individual to acquire a specific disease or phenotype. Although PRS loses the ability to pinpoint a single gene or mutation as causal for disease, studies have used PRS approaches to reach predictive powers similar to those seen in much more penetrant single-gene-driven diseases, including in coronary artery disease [319]. This risk stratification strategy is helpful for disease prediction and precision medicine and helps to better evaluate the effectiveness of treatment strategies by stratifying patients into risk groups. A major limitation of PRS is its reliance on the presence of known polymorphisms that are associated with disease and numerous groups have highlighted the difficulty of applying PRS appropriately to understudied populations outside of European ancestries [320]. Some research groups have begun to apply the principles of PRS to study individuals based on variations in mitochondrial haplotype. In these scenarios, the mitochondrial haplotype proved to be a synergistic factor that influences phenotypic diversity and disease progression as seen in Alzheimer’s Disease [321] and obesity [322]. Efforts to apply mitochondrial PRS to the cardiovascular system have been largely lacking and infrequently studied by other groups such as inclusion of mitochondrial genes as a driver in PRS estimation of Torsade de Pointes in the context of patients taking antipsychotic medication for treatment of schizophrenia [323]. Truby (2021) specifically used mitochondrial PRS to predict HF in European population, based on nuclear genes affecting synthesis of long chain acyl carnitine [324]. However, studies based on mitochondrial encoded genes, wider populations (non-European) and inclusive of several cardiometabolic disease traits are necessary. Of late, mtDNA copy-number (mtDNAcn) has become *de facto* biomarker of healthy heart and computational methods have utilized this to generate ‘mitoscore’ based PRS for the ESTHER study [325]. This has clearly associated novel risk loci with variation in mtDNAcn, but more research is needed to thoroughly link these scores to downstream cardiovascular events in a wider population and mtDNA variants.

### Detecting heteroplasmy with new techniques

Cardiovascular disease is the most common aging-associated condition, with mitochondrial dysfunction widely recognized as its root cause [326]. Within each cell, multiple copies of mtDNA exist. As we age, the accumulation of ROS partially mutates the mtDNA, leading to a mix of unaltered and mutated forms within the same cell termed ‘heteroplasmy.’ Over time, with increased heteroplasmy, the heart cell’s ability to produce energy diminishes, failing to meet the organ’s demands as mutated mtDNA becomes predominant. This process is accompanied by disrupted mitochondrial dynamics and reduced mitophagy, resulting in the buildup of ROS-generating mitochondria. This creates a vicious cycle, contributing to heart failure. Mitochondrial heteroplasmy has been linked to pathogenic phenotypes in disease based on their frequency and function (synonymous vs. non-synonymous) [327]. However, the majority of research done on heteroplasmy has relied on modest estimation of total heteroplasmic mitochondrial content, rather than the actual effects of different variants seen within the heteroplasmic heart. This is because classic means of studying mtDNA heteroplasmy has been limited to PCR or bulk sequencing techniques that have not offered insights into the variability present between individuals. One approach to this issue is to saturate the space through massively parallel sequencing approaches such as performed by Zhang (2012), in which researchers combined long-range PCR and parallel sequencing methods to detect and quantify heteroplasmy with 100% sensitivity from human samples with known mutations [328]. However, the heteroplasmy in terms of sub-population, and distinguishing micro (within single mitochondria) and macro (within a cell) heteroplasmy remains unresolved. Recently, steps have been taken to creatively overcome this issue through sophisticated analysis approaches. For instance, researchers developed methods combining enzyme histochemistry and single-cell laser microdissection to study mtDNA within individual skeletal muscle fibers by sequencing [329]. Nevertheless, deciphering results in most of the sequencing approaches can prove difficult as they are hindered by NUMT from the nDNA. More recently, single-cell mitochondrial sequencing has helped to establish cell lineages in hematopoiesis and could be potentially useful in the future for establishing protocols to study the spatial distribution of somatic mutations and heteroplasmy in the heart [330, 331]. More research is needed on this topic, due to its outsized impact on cardiovascular phenotypes.

## Conclusions and call for research

In this review, we have set out to describe the current state of the field in terms of understanding the role of mitochondria in cardiovascular health. The vast research in the last decade has proved beyond the fact that the mitochondria no longer are confined to the powerhouse analogy and have become signal processors of the cell [332]. We have described how mitochondria are vital to cardiac development, homeostasis of a physiologically healthy heart, the ways in which the mitochondria are affected by pathological remodeling of the heart and how they can, in their own way, contribute to disease progression. Finally, we have highlighted the advent of new computational approaches to better understand the role of mitochondria in phenotype variability. A common theme of our review is the repeated observation that mitochondria are often overlooked as a driver of phenotype, either ignored or, in some cases, deliberately filtered out from the data in order to ‘clean’ it. Recent advances in the field have shown that mitochondria are associated with many phenotypes (spatially distinct, genetically heterogenous, and functionally specialized) and are a key player in CVD. It is vitally important that these ‘discarded’ castoffs of the filtering process for, for example, RNAseq analyses are recovered and studied by interested groups as these existing datasets represent large amounts of untapped but crucially relevant data.

## Supplementary Information

Supplementary Material 1.

## Data Availability

No datasets were generated or analysed during the current study.
