# Supplementary material for: The multifaceted role of mitochondria in cardiac function: insights and approaches
Source: Cell Commun Signal. 2024 Oct 29;22:525. doi: 10.1186/s12964-024-01899-x (PMC11523909; doi:10.1186/s12964-024-01899-x)
Supplement: Supplementary file 1 — Supplementary Material 1. [file 12964_2024_1899_MOESM1_ESM.docx]

**Additional table 1:** Studies integrating mitochondrial dysfunction with cardiac structure and function in CVD. The impact of disease on the heart function and mitochondria is summarized rather than the intervention.

| **S.No.** | **Mitochondrial dysfunction in heart** | **Functional Consequence on heart** | **Structural consequence on heart** | **Cardiac Disease condition** | **Intervention** | **Species**  **/Model** |
| --- | --- | --- | --- | --- | --- | --- |
| 1 | *Structure*-Reduction in surface area, perimeter, lower density and integrity with swelling, loss in cristae structure and disrupted inner /outer membranes,  *Respiration*- Lower RCR, Copy-number  *Dynamics*- downregulation of genes encoding for proteins involved in fusion (Mfn1/2) and fission (*Drp1 and Fis1*).  Elevated SOD2  [1] | ↑posterior wall thickness (indexed), smaller cavity, and ↓end-diastolic volume and cardiac output in OIC versus control | ↑intracardiac lipid deposition No differences between control and OIC groups in terms body weight, heart weight, and heart/body weight ratio, | Cardiomyopathy after pre-term birth | Neonatal hyperoxia by oxygen from postnatal day3 to 10 | Male SD rats |
| 2 | *Structure-* swollen with reduced ΔΨm and undergoing early cell apoptosis  *Respiration*-Reduced basal respiration, maximal respiration, and ATP production due to doxorubicin  [2] | ↓HR, %EF, %FS, dp/dt, | H&E showed disordered cell arrangement, TUNEL stain showed apoptosis, decreased ΔΨm also predicted early cell apoptosis by JC1 dye | Doxorubicin induced cardiotoxiicty | Corosolic acid | C57 BL/6J mice and AMPKα2 knockout mice |
| 3 | *Structure* - classic ragged-blue fibers in muscle biopsy revealed with patches of pale cytochrome c oxidase regions [3] | Dilated cardiomyopathy related death of younger sibling | Elevated creatine kinase and exercise intolerance | AV block | TOP3A mutation | Case-study |
| 4 | *Structure* - IRI in diabetic rats caused myofilament and mitochondrial damage with vacuolated mitochondria.  *Oxphos*-ATP levels and complex-I to IV activities were reduced [4] | IRI in diabetic rats  ↓%EF, %FS, and stroke volume | Masson’s stain showed elevated fibrosis and leukocyte infiltration. IHC showed elevated 4HNE levels | Ischemia-reperfusion | GYY4137 (H2S donor), and sevoflurane | Diabetic rats |
| 5 | *Structure* – isolated cardiomyocytes stained with Mito trackers dyes showed elevated mitochondrial density and swelling due to HFpEF which were mitigated by sotagliflozin.  *Dynamics*-increased mitochondrial fission and reduced Ca^+2^ uptake was observed in isolated LA cardiomyocytes due to glucose starvation and was restored in sotagliflozin treatment [5, 6] | ↑beta hydroxybutyrate accumulation | LA enlargement | HFpEF | High caloric diet and SGLT-1&2 inhibitor sotagliflozin | Wistar and ZSF-1 obese rat |
| 6 | *Structure* – loss of mitochondrial pearl string appearance in T2DM hearts. Cristae lacked cohesion and increased size (area) was observed with lower cristae volume density. Exaggerated Ca2+-induced mitochondrial swelling was noted.  *Respiration*-reduced OCR and Oxygen consumption.  *Dynamics*-No significant change in mitochondrial fusion proteins (mitofusin-1, mitofusin-2, and OPA1), proteins involved in mitochondrial fission (DRP-1, and MFF), and mitochondrial-specific signaling proteins (VDAC1 and TOM20). Only upregulation of STAT3 was noted with Morphine treatment in T2DM [7] | T2DM resulted in  *↓*%FS, %EF and mean velocity of circumferential fiber shortening (Vcf), unchanged LVIDd, with widened LVIDs indicating compromised systolic contractile function.  ↑intraventricular septal wall thicknesses in diastole (IVSd) and ↓relative wall thickness (LV radius/wall thickness ratio, R/Th) when compared to controls demonstrating hypertrophy in  In exvivo model, Ischemic insult of T2DM heart ↑diastolic pressure, ↓LVDP | T2DM resulted in increased picrosirius red staining. In exvivo exaggerated LDH release resulted in hyper contracture and stunning | Diabetes and ischemia-reperfusion injury (Exvivo) | Morphine  STZ | High fat diet induced T2DM using STZ |
| 7 | *Structure*- Prediabetic mice show skeletonized mitochondria with holes, reduced distance between sarcoplasmic reticulum and mitochondria, decrease in diameter with increased roundness and density.  *Dynamics*-increased mitochondrial calcium and fission. *Oxphos*- Uncoupled ETC, [8] | Confocal imaging of contractile activity of isolated cardiomyocytes show sparks (white arrows), waves, and spontaneous contraction in prediabetic mice | Study was limited to evaluation of ultrastructure of mitochondria in heart | Prediabetic | High fructose diet | C57 BL6 Transgenic mice expressing CaMKII inhibitor peptide-AC3-I |
| 8 | *Structure*- abnormal mitochondrial structure within cardiomyocytes of two siblings with Inherited loss of C1QBP/p32 (a nuclear encoded mitochondrial protein) [9] | Ventricular fibrillation | Cardiomyopathy and left ventricular hypertrophy | Inherited loss of C1QBP/p32 is associated with recessive cardiomyopathy, ventricular fibrillation, and sudden death in early life |  | Human compound heterozygous variants in C1QBP (p.Thr40Asnfs*45 and p.Phe204Leu) in children |
| 9 | *Structure*- marked degeneration of the mitochondria with elevated anti-mitochondrial M2 antibody levels [10] | Cardiac dilatation with diffuse hypokinesis and 30% ↓%EF, Ventricular tachyarrhythmia after 5 year | endomyocardial biopsy showed interstitial fibrosis and cardiomyopathy associated with AMA-positive myositis Anti-mitochondrial antibody | Atypical inflammatory myopathy caused by circulating Anti-mitochondrial antibody (AMA) | NA | Case report |
| 10 | Mitochondria showed elevated VDAC, Sit3 and reduced Cytc-C levels in isolated fractions [11] | In comparison to day 1, 4 weeks of chronic volume overload  ↑LV-chamber dimensions, LV end-systole volume and diastole volume, and LV mass | Hypertrophy and fibrosis noted from Massons, WGA stain and ↑heart wt./Body wt. ratio | Cardiac remodeling secondary to chronic volume overload by aortocaval shunt | Antibody SDS3 | Mice |
| 11 | *Structure*-Hyperglycemia caused vacuolation due to cristae degeneration and marked swelling.  *Dynamics*-Reduced expression of autophagy genes and mtDNA content noted.  Patient hearts had reduced expression of miR-144 [12] | Hyperglycemia caused significant ↓%EF, %FS, E/A ratio, and LV mass. ↑E/e′ ratio, LVIDd and LVIDs. | After 8 weeks hyperglycemia resulted in ↓body weight, and ↑fibrosis (Massons) but no difference in CM size was noted (WGA stain).  Heart weight/body weight ratio showed no significant change while reduced heart weight/tibia length ratio was noted. | Hyperglycemia | STZ induction for 5 days | Male C57BL/6 mice and T2DM patients with cardiac dysfunction and w/o CAD |
| 12 | *Structure*- loss of cristae, swelling, accumulation of 8-oxo-dG indicative of mtDNA damage caused by IRI  *Oxphos*-reduced respiratory capacity, no change in mitochondrial complex protein subunit expression,  *Dynamics*- reduced ACO2, MFN1&2 protein expression, reduced gene expression pf PGC but not TFAM [13] | IRI in non-transgenic mice resulted in ↓%EF and FS which was not the case in Trx overexpressed mice | ↑infarct size in aged non-transgenic mice compared to mutant ones | Ischemia-reperfusion injury | Aging | Aged mice C57BL6: Thioredoxin overexpressing mice, Inactive Trx-mutant overexpressing mice, and non-transgenic litters |
| 13 | *Structure*- irregular, swollen, disordered with broken or disappeared cristae, and vacuolations, higher autophagosomes with residual mitochondria,  *Dynamics*- Higher protein expression of PINK1, PARKIN, and LC3-II in angiotensin group  Lower mitochondrial membrane potential (MMP),  [14] | ↓ %EF, ↑LVPWD,  No difference in LVEDD, LVSDD | ↑HW/BW ratio, fibrosis, and apoptotic cells in myocardium by Annexin-V/PI stain | Hypetrophy induced by angiotensin-II | PINK1/PARKIN role in autophagy observed in hypertrophy | C57BL6 mice Angiotensin-II induced cardiac hypertrophy |
| 14 | *Structure*- fragmented mitochondria with areas of autophagolysis and decrease in average mitochondrial density *Respiration*-Impaired mitochondrial state-3 and state-4 respiration,  [15] | ↓%EF, SV index, %FS and marked LV systolic and diastolic enlargement | ↑scar tissue and fibrosis | Myocardial infarction | Cardiac overexpression of the S100A1 | Rat model of LAD ligation and evaluated after 10 weeks |
| 15 | *Structure*- Increased size in flow cytometry and TEM images noted.  Membrane potential and internal complexity remained unchanged.  *Respiration*- complex III activity and state-3 respiration reduced following exposure.  [16] | ↓%EF and %FS  PW doppler showed diastolic dysfunction and ↑LA pressure  Stress strain speckle tracking revealed ↑diastolic radial strain | Not performed | Titanium dioxide nanoparticle exposure effect on heart | Nano-TiO_2_ P25 powder | FVB mice with miRNA-378 knockout |
| 16 | *Dynamics*-mtDNA copy number and mitofilin protein were lower in hemodialysis patients compared to control. Among those with and w/o LVH, significantly lower in cases with LVH [17] | Significantly ↑Left ventricular mass index (LVMI) vs non LVH undergoing dialysis.  No change in BP | Not performed | Left ventricular hypertrophy in hemodialysis | - | Patients undergoing hemodialysis |
| 17 | *Structure*- Cristae appeared vague, edematous, and broken in portions of the infarct tissue. The matrix appeared blurry with broken mitochondria. myofibril was unevenly thick with indistinguishable sarcomere. Most of the myofilaments were destroyed and dissolved. Also, part of the disordered intercalated discs had poor continuity [18] | ↓%EF, enlargement of LVESId, LVEDId,, ST elevation in ECG,  No difference in heart rate | ↑left ventricle weight index, focal necrosis, karyopyknosis, karyorrhexis and dilation of capillaries. Inflammatory cell infiltration and necrosis were noted in epicardium. Massons staining revealed massive fibrosis with hypertrophy | Acute myocardial infarction | Paeoniflorin | Wistar rats undergoing LAD ligation |
| 18 | ***Structure-* post treatment, the mitochondria from sub-sarcolemmal, interfibrillar and perinuclear regions showed no difference in morphology in response to IRI. S**welling and disorganization of cristae, area, perimeter, and Feret-diameter normalized with increased roundedness after treatment.  *Oxphos*-No significant reduction in RCR but ATP production reduced by half [19] | IR resulted in ventricular premature beats (VPBs), ventricular tachycardia (VT), Ventricular fibrillation (VF) | Study was limited to evaluation of ultrastructure of mitochondria in heart | Ischemia-reperfusion injury | Sodium nitrite | Dogs |
| 19 | *Structure*-swollen mitochondria with less dense cristae. PAH resulted in fragmentation of mitochondrial network with reduced membrane potential.  *Dynamics*-Increased mitochondrial Drp1 compared to cytosol. Reduced aconitase activity noted.  [20] | ↓PAAT, CO, RVFW thickening and TAPSE, ↑Fulton index, reduced distance run on treadmill. | Microvascular rarefaction | Monocrotaline induced Pulmonary arterial hypertension model with isolated IRI in Langendorff | Midvi-1 | SD rat |
| 20 | *Structure*-COX negative cells observed in staining **[21]** | Holter-ECG recordings in freely moving transgenic mice demonstrated higher rates of PVCs and AV-blocks after MI, episodes of accelerated junctional rhythm noted. In transgenic mice there was increased probability of Ventricular Tachyarrhythmia-induction | Sirius red and H&E stain found no difference in fibrosis and structural changes in control and transgenic mice | Myocardial cryoinfarction | mtDNA deletion | Mouse- transgenic to accelerated accumulation of mtDNA deletions (K320E-Twinkle^Myo)^ |
| 21 | *Structure*- Fragmented with vacuolar degeneration. The **intermyofibrillar mitochondria** were abnormally aligned with the adjacent sarcomeres and were disrupted with loss of mitochondria mass.  *Oxphos*-Reduced complex IV activity and uncoupling of ETC with TCA cycle.  *Dynamics*-Elevated Mitochondrial fission by reducing p-DRP1 levels.  *Gene expressions*-In adult cardiomyocyte, upregulation of the apoptotic marker Puma, the autophagy-related genes LC3-1 and Lamp2, and the atrophy gene atrogin-1, but not Murf1 or SOD2 was noted [6, 22, 23] | Prominent diastolic dysfunction and subtle systolic dysfunction  PV loop analysis showed elevated LV maximal pressure (LVEDP) and chamber stiffness along with reduced heart rate | ↑HW/BW ratio and LV weight-to-body weight ratios,  LV interstitial and perivascular fibrosis noted | HFpEF | FOXO3a regulation of mitochondria in HF. Alleviation of HF using gene delivery of dominant-negative FX3a | Ascending aortic banding in rats |
| 22 | *Structure*-Damaged cristae observed in IFM of diabetic mice.  *Respiration*- Respiratory activity declined due to diabetes in IFM but not in SSM.  *Oxphos*- In diabetic hearts only IFM, complex-I to V activities declined but not in SSM. No difference in BNPAGE protein expression of complexes across all groups IFM/SSM. Diabetic IFM showed reduced membrane potential but not SSM  *Dynamics*- no change in expression of Mfn1, Mfn2, OPA1, CHCHD3, and Drp1 among IFM and SSM. mtDNA content showed no difference in mitofilin-TG mice compared to control.  [24] | ↓%EF,%FS and cardiac output in type 1 diabetic heart.  No significant differences in stroke volume, or volumes at systole and diastole, diameter at systole and diastole, heart rate between any groups. | Diabetic animals have significant ↓HW/BW ratio | Diabetic heart | Induced with diabetes using multiple low dose streptozotocin | FVB mouse ad Mitofilin transgenic FVB mouse |
| 23 | *Structure-*Mitochondria were reduced in size and more abundant per square μm in WT-TAC hearts.  *Dynamics*-Expression of fission marker Drp1 was very high due to pressure overload.  *Oxphos-*Genes encoding OXPHOS were significantly downregulated, and protein expression of complex-I to V were significantly downregulated with decline in ATP levels in WT-TAC heart [25] | No change in heart rate, IVS thickness, due to TAC in both WT and fat-1 mice. Significantly ↑LVID and ventricular volume. ↓%EF and %FS | ↑cardiomyocyte size and fibrosis | Dilated cardiomyopathy | Pressure overload in mice by transverse aortic constriction | Transgenic cardiomyocyte specific FATP-1 mouse. |
| 24 | *Structure*- fracture of mitochondrial edges and membranes was noted apart from oedema.  *Respiration-*Isolated mitochondria showed reduced oxygen consumption, calcium retention and elevated ROS generation [26] | Ventricular fibrillation, ↑HR, ↓MAP (monophasic action potential), LV dP/dt_Max_ and mean blood pressure | ↑ischemic area, Low granulation density as noted by enzymatic loss of SDH, ATPase and Phosphorylase activity in myocardium. Pyknotic nucleus, high variability in the membrane thickness, irregular accumulation of chromatin in the nucleus, and thickening of capillary walls | Acute coronary ischemia | Trimetazidine (antianginal) | Pigs |
| 25 | Decrease in phosphocreatine-to-ATP ratio in ventricles [27] | Reduced time of ventricular fibrillation | Study was limited to evaluation of ultrastructure of mitochondria in heart | Ischemia induced ventricular fibrillation | Ivabradine and propranolol | Pigs |
| 26 | *Structure*-Swollen mitochondrial with disorganized cristae and reduced matrix density [28] | decreases in recoveries of LVDP, RPP, dp/dt max and dp/dt min and a remarkable  increase in LVEDP after reperfusion | ↑infarct size and apoptosis (TUNEL stain) | Ischemia reperfusion injury | Xenon, diazoxide and 5HD | Rabbit |
| 27 | *Structure*- prominent swelling or disruption of mitochondria, intracellular or perinuclear edema, and sarcoplasmic vacuoles resulting from dilation of sarcoplasmic reticulum. However, marked loss of myofilaments and alterations of characteristic sarcomeric structure were not observed [29] | 4-week rapid pacing caused global  ↑Dd/Ds and E/E′,  ↓%EF, ESWT, and EDWT.  Regional systolic function by speckle-tracking showed ↓peak systolic radial, circumferential, transverse, and longitudinal strains | Cardiomyopathy hearts had thin LV walls with large LV cavity, increased fibrosis, mean transverse cellular diameter, and vascular density, all of which were reversed in ONO1301SR treated ones | Rapid pacing induced dilated cardiomyopathy | ONO1301SR (prostacyclin agonist) | Beagle Dog |
| 28 | ***Structure*-Exvivo atrial tissues from patients with AF showed impaired mitochondrial structure and respiration. Similar effects were noted when tissues were rapidly paced for 24 h exvivo [30]** | Atrial fibrillation | Study was limited to evaluation of ultrastructure of mitochondria in heart | Atrial fibrillation | Exvivo treatment with verapamil, apocynin, resveratrol and Olmesartan | Human |
| 29 | *Structure***-**Isolated mitochondria showed most of the inner and outer membranes were disrupted, displaying destroyed cristae formation and a swollen matrix. *Respiration*-Reduced respiratory control index [31] | Limited recovery of LVDP and dP/d*t* post reperfusion | Study was limited to evaluation of ultrastructure of mitochondria in heart | Ischemia-reperfusion injury | Geranylgeranyl acetone (antiulcer) | Rat |
| 30 | *Structure*-Swollen mitochondria with less cristae density  *Respiration*-reduced oxygen consumption and RCR [32] | ↓%EF and %FS. ↑LVEDD, LVESD and HR | ↑infarct size and TUNEL positive nuclei with elevated troponin levels | Ischemia-reperfusion injury | Hydrogen sulfide | C57BL6/J mice |

HR- heart rate, EF-ejection fraction, FS- fractional shortening, dp/dt- rate of change of ventricular pressure, AV- atrioventricular, IRI- ischemia-reperfusion injury, IHC- immunohistochemistry, LA-left auricle, HFpEF- heart failure with preserved ejection fraction, T2DM- diabetes mellitus type2, OCR- oxygen consumption ratio, LVIDs- Left ventricular internal diameter at systole, LVIDd- Left ventricular internal diameter at diastole, IVSd- interventricular septal diameter, STZ- streptozotocin, LVDP- left ventricular developed pressure, RPP- rate pressure product, ΔΨm- mitochondrial membrane potential, CAD- coronary artery disease, LVPWD- left ventricular posterior wall diameter, HW- heart weight, BW- body weight, LVEDD- Left ventricular end-diastolic diameter, LVSDD- Left ventricular end-systolic diameter, SV- stroke volume, LAD- left anterior descending artery, TEM- transmission electron microscopy, CO- cardiac output, RVFW- right ventricular free wall, TAPSE- Tricuspid annular plane systolic excursion, PV loop- pressure-volume loop, BNPAGE- blue native polyacrylamide gel electrophoresis, IFM- inter fibrillar mitochondria, SSM- Sub-sarcolemmal mitochondria, TAC- transverse aortic constriction, MAP-mean arterial pressure.

**References**

1. Ravizzoni Dartora D, Flahault A, Pontes CNR, He Y, Deprez A, Cloutier A, Cagnone G, Gaub P, Altit G, Bigras JL, et al: **Cardiac Left Ventricle Mitochondrial Dysfunction After Neonatal Exposure to Hyperoxia: Relevance for Cardiomyopathy After Preterm Birth.** *Hypertension* 2022, **79:**575-587.

2. Che Y, Wang Z, Yuan Y, Zhou H, Wu H, Wang S, Tang Q: **By restoring autophagic flux and improving mitochondrial function, corosolic acid protects against Dox-induced cardiotoxicity.** *Cell Biology and Toxicology* 2022, **38:**451-467.

3. Primiano G, Torraco A, Verrigni D, Sabino A, Bertini E, Carrozzo R, Silvestri G, Servidei S: **Novel TOP3A Variant Associated With Mitochondrial Disease: Expanding the Clinical Spectrum of Topoisomerase III Alpha-Related Diseases.** *Neurol Genet* 2022, **8:**e200007.

4. Zhang J, Cai X, Zhang Q, Li X, Li S, Ma J, Zhu W, Liu X, Wei M, Tu W, et al: **Hydrogen sulfide restores sevoflurane postconditioning mediated cardioprotection in diabetic rats: Role of SIRT1/Nrf2 signaling-modulated mitochondrial dysfunction and oxidative stress.** *Journal of Cellular Physiology* 2021, **236:**5052-5068.

5. Bode D, Semmler L, Wakula P, Hegemann N, Primessnig U, Beindorff N, Powell D, Dahmen R, Ruetten H, Oeing C, et al: **Dual SGLT-1 and SGLT-2 inhibition improves left atrial dysfunction in HFpEF.** *Cardiovasc Diabetol* 2021, **20:**7.

6. Chaanine AH, Kohlbrenner E, Gamb SI, Guenzel AJ, Klaus K, Fayyaz AU, Nair KS, Hajjar RJ, Redfield MM: **FOXO3a regulates BNIP3 and modulates mitochondrial calcium, dynamics, and function in cardiac stress.** *Am J Physiol Heart Circ Physiol* 2016, **311:**H1540-h1559.

7. Zemljic-Harpf AE, See Hoe LE, Schilling JM, Zuniga-Hertz JP, Nguyen A, Vaishnav YJ, Belza GJ, Budiono BP, Patel PM, Head BP, et al: **Morphine induces physiological, structural, and molecular benefits in the diabetic myocardium.** *Faseb j* 2021, **35:**e21407.

8. Federico M, Zavala M, Vico T, López S, Portiansky E, Alvarez S, Abrille MCV, Palomeque J: **CaMKII activation in early diabetic hearts induces altered sarcoplasmic reticulum-mitochondria signaling.** *Sci Rep* 2021, **11:**20025.

9. Webster G, Reynolds M, Arva NC, Dellefave-Castillo LM, McElligott HS, Kofman A, Laboski A, Magnetta D, George AL, Jr., McNally EM, Puckelwartz MJ: **Mitochondrial cardiomyopathy and ventricular arrhythmias associated with biallelic variants in C1QBP.** *Am J Med Genet A* 2021, **185:**2496-2501.

10. Hasegawa Y, Izumi D, Kashimura T, Minamino T: **Life-threatening ventricular arrhythmia and left ventricular dysfunction associated with anti-mitochondrial antibody-positive myositis: a case report.** *Eur Heart J Case Rep* 2021, **5:**ytab469.

11. Cohen L, Sagi I, Bigelman E, Solomonov I, Aloshin A, Ben-Shoshan J, Rozenbaum Z, Keren G, Entin-Meer M: **Cardiac remodeling secondary to chronic volume overload is attenuated by a novel MMP9/2 blocking antibody.** *PLOS ONE* 2020, **15:**e0231202.

12. Tao L, Huang X, Xu M, Yang L, Hua F: **MiR-144 protects the heart from hyperglycemia-induced injury by regulating mitochondrial biogenesis and cardiomyocyte apoptosis.** *Faseb j* 2020, **34:**2173-2197.

13. Subramani J, Kundumani-Sridharan V, Das KC: **Thioredoxin protects mitochondrial structure, function and biogenesis in myocardial ischemia-reperfusion via redox-dependent activation of AKT-CREB- PGC1α pathway in aged mice.** *Aging (Albany NY)* 2020, **12:**19809-19827.

14. Fan G, Chen MJ, Wei J: **Involvement of phosphatase and tensin homolog-induced putative kinase 1/Parkin-mediated autophagy in angiotensin II-induced cardiac hypertrophy in C57BL/6 mice.** *J Int Med Res* 2020, **48:**300060519896143.

15. Katz MG, Gubara SM, Hadas Y, Weber T, Kumar A, Eliyahu E, Bridges CR, Fargnoli AS: **Effects of genetic transfection on calcium cycling pathways mediated by double-stranded adeno-associated virus in postinfarction remodeling.** *J Thorac Cardiovasc Surg* 2020, **159:**1809-1819.e1803.

16. Hathaway QA, Durr AJ, Shepherd DL, Pinti MV, Brandebura AN, Nichols CE, Kunovac A, Goldsmith WT, Friend SA, Abukabda AB, et al: **miRNA-378a as a key regulator of cardiovascular health following engineered nanomaterial inhalation exposure.** *Nanotoxicology* 2019, **13:**644-663.

17. Wu QS, He Q, He JQ, Chao J, Wang WY, Zhou Y, Lou JZ, Kong W, Chen JF: **The role of mitofilin in left ventricular hypertrophy in hemodialysis patients.** *Ren Fail* 2018, **40:**252-258.

18. Chen H, Dong Y, He X, Li J, Wang J: **Paeoniflorin improves cardiac function and decreases adverse postinfarction left ventricular remodeling in a rat model of acute myocardial infarction.** *Drug Des Devel Ther* 2018, **12:**823-836.

19. Demeter-Haludka V, Kovács M, Petrus A, Patai R, Muntean DM, Siklós L, Végh Á: **Examination of the Role of Mitochondrial Morphology and Function in the Cardioprotective Effect of Sodium Nitrite Administered 24 h Before Ischemia/Reperfusion Injury.** *Front Pharmacol* 2018, **9:**286.

20. Tian L, Neuber-Hess M, Mewburn J, Dasgupta A, Dunham-Snary K, Wu D, Chen KH, Hong Z, Sharp WW, Kutty S, Archer SL: **Ischemia-induced Drp1 and Fis1-mediated mitochondrial fission and right ventricular dysfunction in pulmonary hypertension.** *J Mol Med (Berl)* 2017, **95:**381-393.

21. Stöckigt F, Beiert T, Knappe V, Baris OR, Wiesner RJ, Clemen CS, Nickenig G, Andrié RP, Schrickel JW: **Aging-related mitochondrial dysfunction facilitates the occurrence of serious arrhythmia after myocardial infarction.** *Biochem Biophys Res Commun* 2017, **493:**604-610.

22. Chaanine AH, Gordon RE, Kohlbrenner E, Benard L, Jeong D, Hajjar RJ: **Potential role of BNIP3 in cardiac remodeling, myocardial stiffness, and endoplasmic reticulum: mitochondrial calcium homeostasis in diastolic and systolic heart failure.** *Circ Heart Fail* 2013, **6:**572-583.

23. Chaanine AH, Jeong D, Liang L, Chemaly ER, Fish K, Gordon RE, Hajjar RJ: **JNK modulates FOXO3a for the expression of the mitochondrial death and mitophagy marker BNIP3 in pathological hypertrophy and in heart failure.** *Cell Death Dis* 2012, **3:**265.

24. Thapa D, Nichols CE, Lewis SE, Shepherd DL, Jagannathan R, Croston TL, Tveter KJ, Holden AA, Baseler WA, Hollander JM: **Transgenic overexpression of mitofilin attenuates diabetes mellitus-associated cardiac and mitochondria dysfunction.** *J Mol Cell Cardiol* 2015, **79:**212-223.

25. Elezaby A, Sverdlov AL, Tu VH, Soni K, Luptak I, Qin F, Liesa M, Shirihai OS, Rimer J, Schaffer JE, et al: **Mitochondrial remodeling in mice with cardiomyocyte-specific lipid overload.** *Journal of molecular and cellular cardiology* 2015, **79:**275-283.

26. Dehina L, Vaillant F, Tabib A, Bui-Xuan B, Chevalier P, Dizerens N, Bui-Xuan C, Descotes J, Blanc-Guillemaud V, Lerond L, Timour Q: **Trimetazidine demonstrated cardioprotective effects through mitochondrial pathway in a model of acute coronary ischemia.** *Naunyn Schmiedebergs Arch Pharmacol* 2013, **386:**205-215.

27. Vaillant F, Dehina L, Dizerens N, Bui-Xuan B, Tabib A, Lauzier B, Chevalier P, Descotes J, Timour Q: **Ivabradine but not propranolol delays the time to onset of ischaemia-induced ventricular fibrillation by preserving myocardial metabolic energy status.** *Resuscitation* 2013, **84:**384-390.

28. Li Q, Lian C, Zhou R, Li T, Xiang X, Liu B: **Pretreatment With Xenon Protected Immature Rabbit Heart From Ischaemia/Reperfusion Injury by Opening of the mitoKATP Channel.** *Heart, Lung and Circulation* 2013, **22:**276-283.

29. Shirasaka T, Miyagawa S, Fukushima S, Saito A, Shiozaki M, Kawaguchi N, Matsuura N, Nakatani S, Sakai Y, Daimon T, et al: **A slow-releasing form of prostacyclin agonist (ONO1301SR) enhances endogenous secretion of multiple cardiotherapeutic cytokines and improves cardiac function in a rapid-pacing–induced model of canine heart failure.** *The Journal of Thoracic and Cardiovascular Surgery* 2013, **146:**413-421.

30. Bukowska A, Schild L, Keilhoff G, Hirte D, Neumann M, Gardemann A, Neumann KH, Röhl FW, Huth C, Goette A, Lendeckel U: **Mitochondrial dysfunction and redox signaling in atrial tachyarrhythmia.** *Exp Biol Med (Maywood)* 2008, **233:**558-574.

31. Shinohara T, Takahashi N, Kohno H, Yamanaka K, Ooie T, Wakisaka O, Murozono Y, Taniguchi Y, Torigoe Y, Hara M, et al: **Mitochondria are targets for geranylgeranylacetone-induced cardioprotection against ischemia-reperfusion in the rat heart.** *Am J Physiol Heart Circ Physiol* 2007, **293:**H1892-1899.

32. Elrod JW, Calvert JW, Morrison J, Doeller JE, Kraus DW, Tao L, Jiao X, Scalia R, Kiss L, Szabo C, et al: **Hydrogen sulfide attenuates myocardial ischemia-reperfusion injury by preservation of mitochondrial function.** *Proc Natl Acad Sci U S A* 2007, **104:**15560-15565.
